# Synthesis and biological evaluation of *Argemone mexicana*-inspired antimicrobials

**DOI:** 10.3762/bjoc.19.108

**Published:** 2023-09-29

**Authors:** Jessica Villegas, Bryce C Ball, Katelyn M Shouse, Caleb W VanArragon, Ashley N Wasserman, Hannah E Bhakta, Allen G Oliver, Danielle A Orozco-Nunnelly, Jeffrey M Pruet

**Affiliations:** 1 Department of Chemistry, Valparaiso University, 1710 Chapel Dr, Valparaiso, IN 46383, USA; 2 Department of Biology, Valparaiso University, 1610 Campus Dr, Valparaiso, IN 46383, USA; 3 Ivy Tech Community College, 410 E Columbus Dr, East Chicago, IN 46312, USA; 4 Department of Chemistry and Biochemistry, University of Notre Dame, 251 Nieuwland Hall, Notre Dame, IN 46556, USA

**Keywords:** benzylisoquinoline, berberine, chelerythrine, drug discovery, plant-derived antimicrobials

## Abstract

Due to the lack of new antimicrobial drug discovery in recent years and an ever-growing prevalence of multidrug-resistant “superbugs”, there is a pressing need to explore alternative ways to combat pathogenic bacterial and fungal infections. Building upon our previous work in the field of medicinal phytochemistry, the present study is focused on designing, synthesizing, and testing the altered bioactivity of new variants of two original bioactive molecules found in the *Argemone mexicana* plant. Herein, we report upon 14 variants of berberine and four variants of chelerythrine that have been screened against a pool of 12 microorganisms (five Gram-positive and four Gram-negative bacteria, and three fungi). Additionally, the crystal structures of two berberine variants are described. Several berberine variants show enhanced antibacterial activity compared to the unaltered plant-derived molecule. We also report promising preliminary tumor cytotoxicity effects for a number of the berberine derivatives.

## Introduction

The isolation, or creation of novel antimicrobial agents is currently at the forefront of modern healthcare due to the stark decrease in antimicrobial drug development in recent years [1] and due to the increasing rise of superbugs, or microorganisms that are resistant to more than one type of antimicrobial treatment, which are predicted by 2050 to cause 10 million deaths/year [2]. *Staphylococcus aureu*s, for example, is a common opportunistic human pathogen, some strains of which are resistant to multiple antibiotics [3]. Such drug-resistant microbes are especially prevalent in hospital settings, where they are one of the most difficult illnesses to treat [4]. In addition to being a terrestrial cause for concern, antimicrobial-resistant microbes are also a threat to the health of the individuals on the international space station (ISS). According to recent studies, a diverse population of bacteria and fungi, including several opportunistic pathogens, have colonized the ISS [5], and many of these strains have been found to possess antimicrobial resistance genes [6]. With the persistent increase in drug-resistant microbial strains, there is a pressing need to continuously explore new and alternative drug candidates.

Plants naturally produce many compounds that can be used to treat a variety of human diseases. For instance, *Argemone mexicana* has been reported to possess a wide range of biological activities, such as anticancer, antimicrobial, anti-inflammatory, antidiabetic, and antioxidant actions [7]. The *A. mexicana* plant has been used in traditional medicine for centuries [8]. Our previous work explored screening methanol and hexane extracts of various parts of the *A. mexicana* plant (seeds, leaves, inner vs outer roots) for biological activity with the outer root methanol extract showing the highest activity against Gram-positive bacteria as well as inhibitory effects against human colon cancer cells [9]. The quantification of c-MYC (oncogene) and APC (tumor suppressor) mRNA levels helped begin to elucidate how the *A. mexicana* root methanol extract may be affecting colon cancer cells. After chromatographic separations, UPLC–MS, and subsequent nuclear magnetic resonance analysis of the root and leaf methanol fractions, the main bioactive phytochemicals were identified as berberine, chelerythrine, and sanguinarine from this same report [9]. These three compounds are known antimicrobial agents, with a wide variety of biological activities [10–12]. The antimicrobial effects of berberine are often attributed to high binding affinity to DNA, interference with protein biosynthesis, induction of membrane leakage, and affecting GTPase activity in bacteria cell division [12–15]. Recent reports have also pointed to inhibition of the ‘filamenting temperature-sensitive mutant Z’ (FtsZ) protein [16–17], as well as perturbing carbohydrate metabolism to generate reactive oxygen species that damage the DNA [18], as modes of action for berberine’s antibacterial effects. The antitumor properties of berberine have been attributed to DNA binding, and in particular regulating the activity of telomerase and topoisomerases I and II [15,19–20]. It is evident that the effects of berberine are not tied to any single mode of action. Chelerythrine has also been shown to possess a wide variety of biological activities, such as anticancer, antibacterial, and anti-inflammatory actions [11,21–23]. Similar to berberine, results have demonstrated that the antibacterial activity of chelerythrine can be tied to DNA intercalation and disruption to cell membrane permeability [11,24]. One particular mechanism of action noted for chelerythrine’s antitumor bioactivity is through the inhibition of protein kinase C [25]. Due to the wide range of biological activities for both berberine and chelerythrine, several reports have been made on structural derivatives of these compounds [16,23,26–33]. Given the extreme structural similarity between chelerythrine and sanguinarine, chelerythrine analogs with modifications to the ring substituents can be seen as ubiquitous with sanguinarine analogs.

Our group has been focused on exploring new compounds with antibacterial and antifungal properties, which may serve to ease the strain caused by the ever-growing list of drug-resistant microorganisms. To do this, we are building a library of structural variants of phytochemicals isolated from the *A. mexicana* plant to evaluate against the following 12 microorganisms, which were previously identified as being present on the ISS [5]: five Gram-positive bacteria (*Staphylococcus aureus*, *Bacillus cereus*, *Bacillus subtilis*, *Staphylococcus epidermidis*, *Corynebacterium pseudodiphtheriticum*), four Gram-negative bacteria (*Escherichia coli*, *Proteus mirabilis*, *Enterobacter aerogenes*, *Enterobacter cloacae*), and three fungi (*Saccharomyces cerevisiae, Candida albicans, Penicillium chrysogenum*). The goal of this bioactivity screening of the structural variants is to identify unique selectivity in antimicrobial effects, as compared to the original plant-derived compounds.

## Results and Discussion

**Figure 1:**
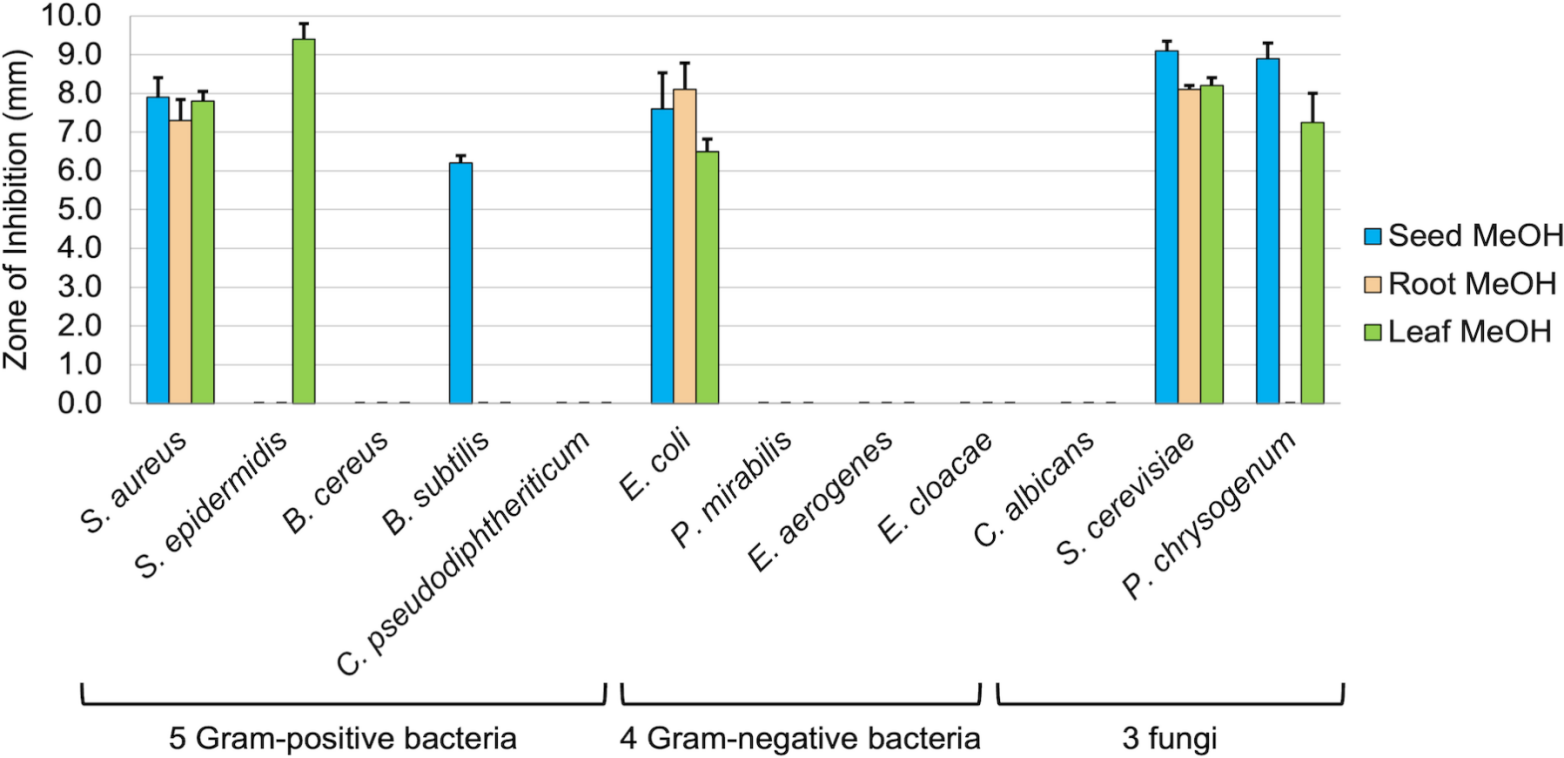
Zones of inhibition for 1 mg of evaporated methanolic (MeOH) extracts from various parts of the *A. mexicana* plant against a panel of 12 microorganisms. The mean zone of inhibition in millimeters is shown with associated standard error (*n* = 5). Vancomycin, streptomycin, and/or fluconazole were used as positive controls, and solvents were used as negative controls and showed no zones of inhibition.

Prior to evaluating the activity of berberine and chelerythrine variants against the full panel of 12 microbes, we first re-assessed the various extracts of the *A. mexicana* plant parts against this same panel, as our original study had only focused on the plant’s activities against six representative microbes [9]. Activities were assessed through a Kirby–Bauer disc-diffusion assay, measuring zones of inhibition in millimeters. An overall summary of the various plant extracts against the complete set of 12 microbes is displayed in Figure 1.

**Scheme 1:**
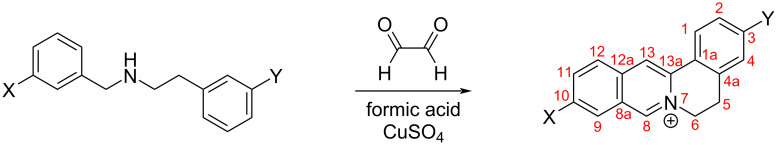
General route to berberine variants, displaying the numbering system for the berberine ring.

Our synthetic work began with the construction of the berberine derivatives. Several synthetic routes to berberine have been reported [28,30,33–34], but by far the most streamlined method involves a copper-promoted Pictet–Spengler-type cyclization with glyoxal, with oxidative aromatization at the 8-position (Scheme 1) [30,35].

**Scheme 2:**
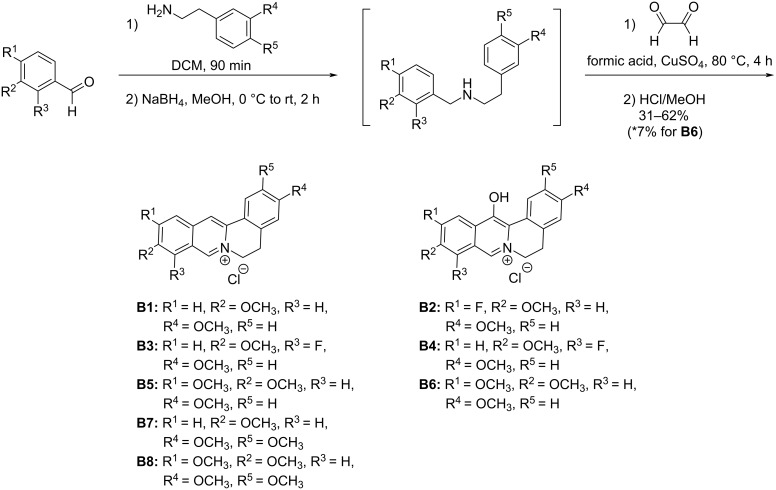
Synthesis of new berberine variants. Reductive amination to a secondary amine was followed by cyclization with glyoxal to provide the desired derivatives. Unexpected oxidation side-product **B2**, **B4**, and **B6** were also isolated from certain reaction mixtures.

**Figure 2:**
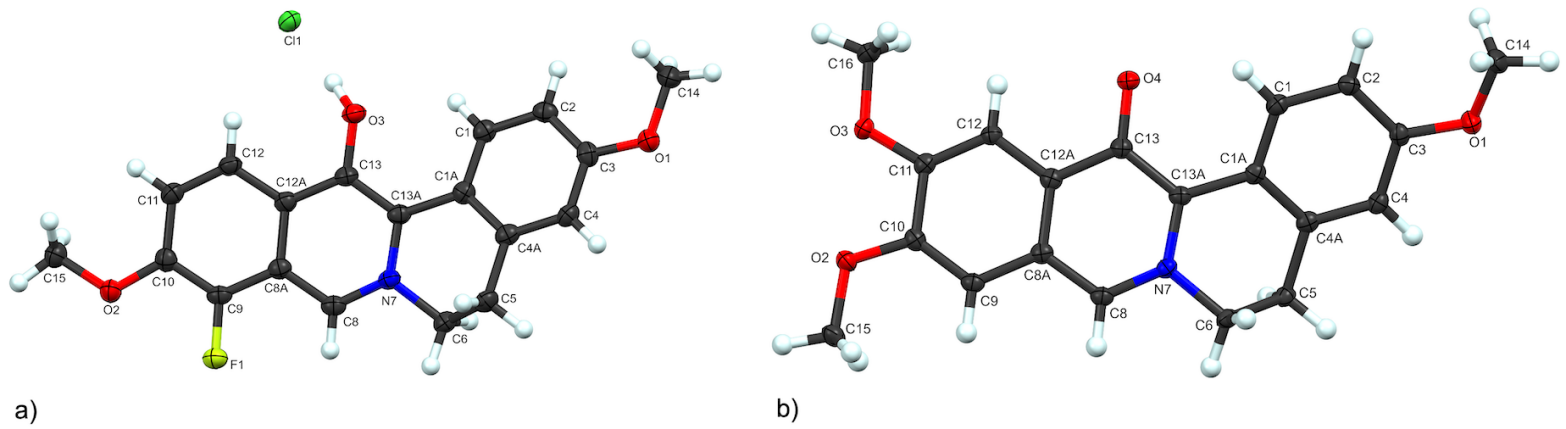
X-ray crystal structures of the oxidation byproducts a) **B4** (CCDC 2271457) and b) **B6** (CCDC 2271458; one molecule selected, second molecule, and solvent omitted for clarity (see Supporting Information File 1 for details).

A recent report suggested a mechanistic role of Cu^2+^ involving C–H activation [36]; however, it is known that this reaction proceeds smoothly to the dihydroberberine in the absence of the copper salt [37]. This suggests the Cu^2+^ may be involved in aiding in the air-oxidation to the fully aromatic berberine core. The prime benefit of the route shown in Scheme 1 is the ease of introducing structural variability, as the precursor is easily generated via reductive amination of a substituted benzaldehyde and a substituted phenethylamine [30]. Thus, a variety of substituted berberine variants were rapidly generated as shown in Scheme 2. Our first variant (**B1**) resulted from the reductive amination of *m*-anisaldehyde with 3-methoxyphenethylamine, followed by cyclization with glyoxal in formic acid. We then wished to slightly perturb the electron density via introduction of fluorine (either at R^1^ or R^3^), but it was at this time that an unexpected result was observed. Following the same conditions which produced **B1**, NMR evaluation of our next product **B2** showed one less aromatic proton than expected and mass spectrometry revealed the presence of an extra oxygen. It was initially thought this unexpected oxidation had occurred at position-8, leading to an 8-oxoberberine variant. However, oxidation at position-8 was questionable (qualitatively) as 8-oxoberberine has been reported as a white solid, while our oxidized product maintained the bright yellow color of berberine [38]. This same unexpected oxidation was observed, to varying degrees, in the production of our next two variants wherein the expected products **B3** and **B5**, respectively were isolated as a mixture with the oxidation side products **B4** and **B6**. The extent of this byproduct formation varied significantly, with exclusive oxidation being observed for **B2**, a near 50:50 ratio of **B3**/**B4**, and the expected **B5** dominating over **B6** in a 9:1 ratio. At this point, the location of this unexpected oxidation was still unclear, and so we grew high quality crystals of **B4** and **B6** to unambiguously determine the structure through X-ray crystallography, which showed the oxidation was in fact occurring at position-13 (Figure 2). A potential mechanistic explanation for the formation of this oxidation byproduct can be found in the Supporting Information File 1.

While unexpected, these oxidative byproducts were still viewed as adding to the structural variability in our berberine series. As the role of Cu^2+^ was believed to assist in the oxidative aromatization of the expected berberine products, we explored minimizing the amount of CuSO_4_, as well as the timing of its addition to the cyclization reaction, hoping to limit the oxidation side reaction. This was indeed beneficial, as the **B3**/**B4** ratio changed from 50:50 to >95% formation of **B3**. Interestingly, all attempts at rerunning the reaction that produced **B2** under these optimized conditions still resulted in exclusive isolation of **B2** with no evidence of the product lacking the oxygen at position-13. Subsequent variants **B7** and **B8** were synthesized as the expected berberine derivatives, without formation of the oxidation byproduct. Of this initial set of berberine derivatives, compound **B8** represents the natural compound pseudopalmatine [39]. The synthesis of this compound has been reported, albeit through alternative methods [33,38,40]. Reports indicated that unlike most other plant-derived protoberberines, pseudopalmatine has had far less pharmacological investigation [39]. Nevertheless, pseudopalmatine has been investigated for RXRα activator activity, and as an up-regulator for both low-density-lipoprotein receptor (LDLR) and insulin receptor (InsR) [33,41]. The compound **B5** was also recently reported and investigated for RXRα activator activity [33]. While these compounds have been reported before, they were still of interest, as neither compound has been thoroughly assessed for its antimicrobial activity.

Our initial pool of berberine variants was screened against 12 microbial organisms (five Gram-positive and four Gram-negative bacteria, and three fungi) to evaluate their antimicrobial activities compared to original berberine, via a Kirby–Bauer test with 0.12 mg of the compound tested per disc (Table 1). It was immediately apparent that the polar substituent at position-13 for variants **B2**, **B4**, and **B6** effectively abolished any activity. This result is consistent with previous reports that showed antimicrobial improvement for variants with hydrophobic substituents at position-13 [26,42]. On the other hand, variant **B1** was significantly more active against Gram-positive bacteria when compared to original berberine (**B**). It was also found **B1** was markedly less potent towards fungi. Though antifungal properties are desirable, selectivity for prokaryotic over eukaryotic cells would be beneficial for a selective antibacterial treatment. A two-tailed T-test analysis (with significance set at *P* ≤ 0.05). revealed that these differences in activity for compounds **B1** vs **B** were statistically significant, apart from *B. subtilis* and *P. chrysogenum* (Supporting Information Information File 1). Much like berberine itself, most of the variants did not show activity against Gram-negative bacteria. However, derivatives **B3** and **B5** did display some Gram-negative activity.

**Table 1:** Kirby–Bauer zones of inhibition for the preliminary berberine variants **B1–B8** compared to unaltered berberine (**B**) as methanol solutions tested against 12 unique microbial species.

Microbe	Mean zones of inhibition (mm)^a^								
	**B** (*n* = 6)	**B1** (*n* = 5)	**B2** (*n* = 3)	**B3** (*n* = 3)	**B4** (*n* = 4)	**B5** (*n* = 5)	**B6** (*n* = 3)	**B7** (*n* = 3)	**B8** (*n* = 3)

*S. aureus*	7.5	12.2	–	9.7	–	10.2	–	6.8	–
*B. cereus*	6.3	8.5	–	–	–	7.1	–	–	–
*B. subtilis*	6.5	6.6	–	7.3	–	–	–	6.3	7.3
*S. epidermidis*	9.0	14.4	–	–	–	6.6	–	11.3	7.7
*C. pseudodiphtheriticum*	6.7	7.4	7.0	8.7	7.4	8.2	–	7.0	–
*E. coli*	–	–	–	6.7	–	–	–	–	–
*P. mirabilis*	–	–	6.7	6.7	–	6.8	–	–	–
*E. aerogenes*	–	–	–	–	–	8.4	–	–	–
*E. cloacae*	–	–	–	9.7	–	10.0	–	–	–
*S. cerevisiae*	15.5	11.4	–	10.7	–	–	–	–	–
*C. albicans*	14.5	9.1	–	14.7	6.5	6.1	–	6.7	–
*P. chrysogenum*	9.0	9.0	–	–	–	6.8	–	–	–

^a^Mean zones of inhibition in millimeters for 0.12 mg of each compound. A dash (–) indicates no measurable antimicrobial effect. Vancomycin, streptomycin, and/or fluconazole were used as positive controls, and solvents were used as negative controls and showed no zones of inhibition.

The zones of inhibition presented in Table 1 were collected using methanolic solutions of compounds, as this maintained consistency with our previous work studying the methanol extracts of the *A. mexicana* plant. However, we recognized some prior literature reports for zones of inhibition for original berberine used a DMSO solution [11]. Thus, we reevaluated a number of variants and berberine itself using DMSO solutions at the same concentrations. Several results showed comparable zones of inhibition to those collected with methanol solutions, with one significant exception. When tested against *S. aureus*, the DMSO solutions were roughly 1.4 times more potent than results with the methanol solution (see Supporting Information File 1). This improved potency was seen for original berberine as well as the variants tested. Variants that were inactive as methanol solutions remained inactive when their DMSO solutions were tested. While this was a notable improvement against *S. aureus*, the general trends in potency were in agreement with those presented in Table 1. Furthermore, some organisms showed a weak zone of inhibition with the DMSO blank. This fact, coupled with the general trends in variable potency matching those seen with the methanol solutions, led us to rely on the inhibitory results for the methanolic samples, as this also better aligned with our prior publication on *A. mexicana*.

**Scheme 3:**
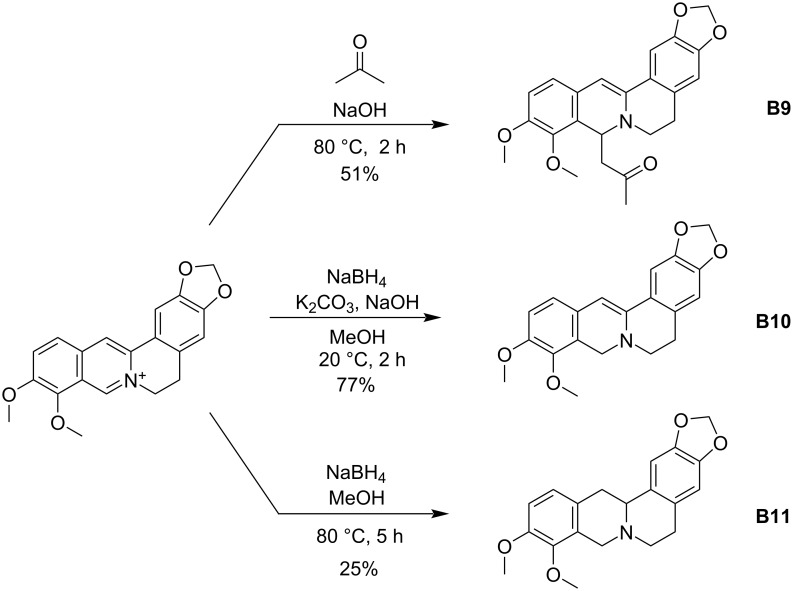
Direct modification of the original berberine structure.

As derivative **B1** was the most promising lead from our original pool of berberine variants, we considered ways to further enhance this activity. We first explored the effects of structural modifications to berberine itself. The cationic iminium within berberine and its derivatives is susceptible to nucleophilic attack [10,14]. Through use of an acetone enolate, as well as partial or full reduction by NaBH_4_, three known berberine variants **B9**–**B11** were prepared (Scheme 3). While the three compounds have previously been prepared, they were synthesized primarily for direct comparison to berberine at the same dose and against the same microbial panel in the present study to ensure confidence in the effects of each modification. It was found that the acetone adduct **B9** and the partially reduced variant **B10** were more potent, while the fully reduced variant **B11** was significantly less active (Table 2). These results suggested the activity of **B1** could be similarly altered by the same modifications to the iminium group.

**Scheme 4:**
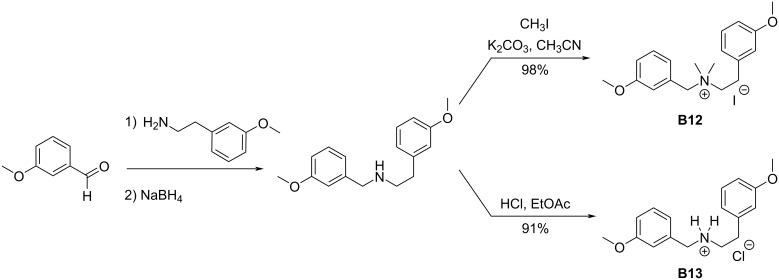
Preparation of non-cyclic charged variants of **B1**.

In addition to assessing how the effects of the above berberine modifications could enhance the activity of **B1**, we also examined the influence of the rigid cyclic structure of compound **B1** by preparing the non-cyclic charged variants **B12** and **B13** (Scheme 4). Our exploration of non-cyclic variants was largely inspired by a recent report that prepared flexible secondary ammonium cations with structural similarity to chelerythrine, wherein the authors did see notable biological potential in these flexible variants [32]. The influence of a fixed charge was also assessed, as protonated compound **B13** can change its charge state depending on local environment, while the methylated derivative **B12** remains a fixed-charge species. While some previous reports have pointed to the cyclic structure as essential for berberine activity, these non-cyclic variants did show some modest activity, albeit typically less active when compared to the **B1** variant. Exceptions to this were the markedly improved activity of compound **B13** towards *C. pseudodiphtheriticum,* as well as its unique Gram-negative activity (Table 2).

**Table 2:** Kirby–Bauer zones of inhibition of the modified variants **B9**–**B14** as tested against 12 unique microbial species.

Microbe	Mean zones of inhibition (mm)^a^					
	**B9** (*n* = 4)	**B10** (*n* = 3)	**B11** (*n* = 3)	**B12** (*n* = 4)	**B13** (*n* = 4)	**B14** (*n* = 5)

*S. aureus*	9.3	12.0	7.3	7.8	7.2	15.1
*B. cereus*	6.5	8.3	–	6.6	6.7	10.0
*B. subtilis*	7.8	10.0	7.0	6.9	12.7	19.2
*S. epidermidis*	–	12.0	9.0	11.8	9.7	30.8
*C. pseudodiphtheriticum*	–	6.3	6.7	6.6	15.7	12.8
*E. coli*	–	6.3	6.7	–	6.8	–
*P. mirabilis*	–	–	–	–	–	–
*E. aerogenes*	–	7.7	–	–	6.7	–
*E. cloacae*	10.0	6.7	–	–	7.5	6.5
*S. cerevisiae*	7.5	14.3	11.7	–	–	7.8
*C. albicans*	9.5	10.7	7.3	–	–	9.4
*P. chrysogenum*	–	12.0	–	–	–	–

^a^Mean zones of inhibition in millimeters for 0.12 mg of each compound. A dash (–) indicates no measurable antimicrobial effect. Vancomycin, streptomycin, and/or fluconazole were used as positive controls, and solvents were used as negative controls and showed no zones of inhibition.

**Scheme 5:**
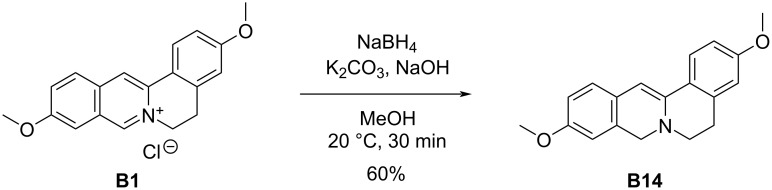
Partial reduction of compound **B1** to **B14**.

**Figure 3:**
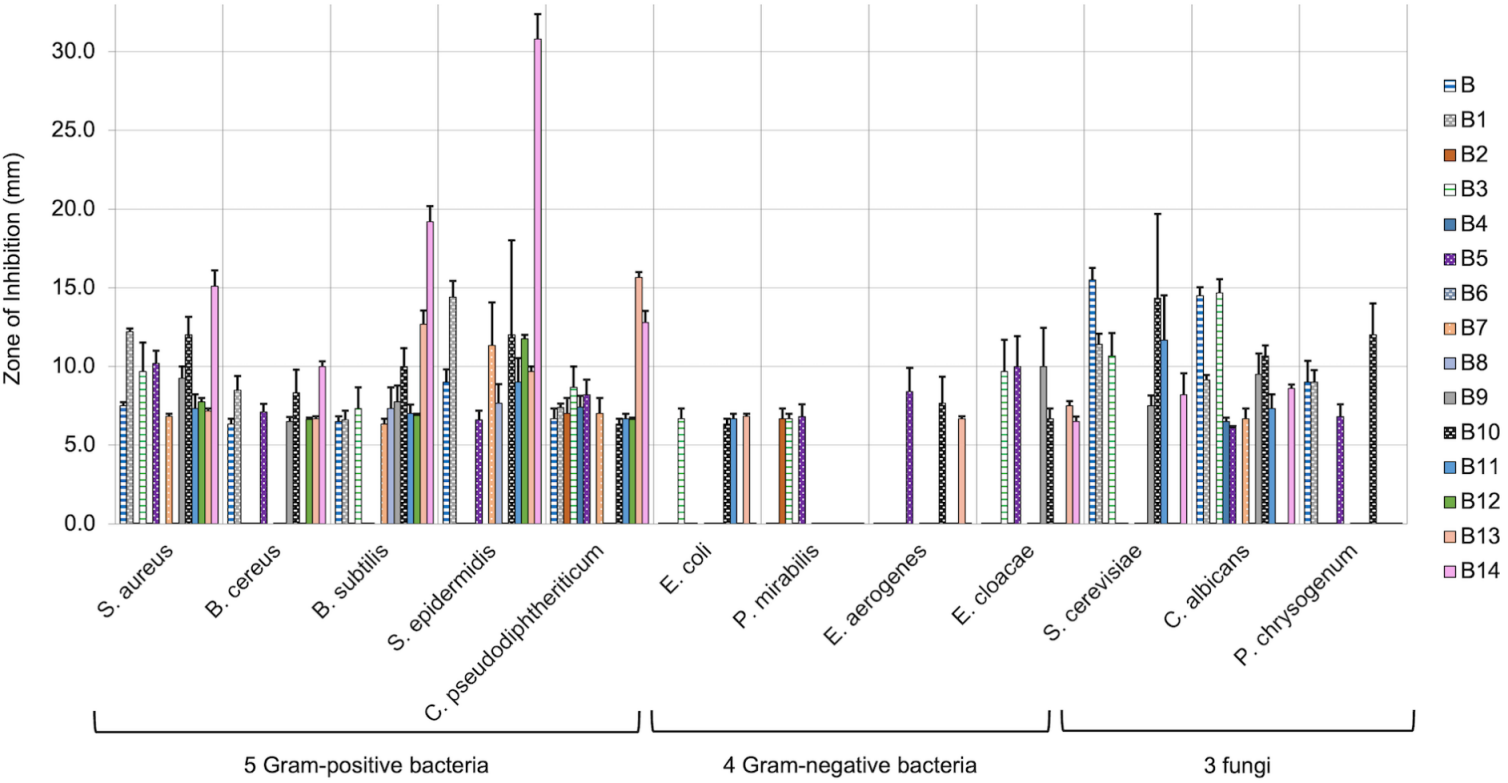
Kirby–Bauer zones of inhibition for all variants **B1**–**B14** compared to original berberine (**B**). Mean zones of inhibition using 0.12 mg of each compound tested against 12 unique microbial species shown with the associated standard error (*n* = 3–6). Vancomycin, streptomycin, and/or fluconazole were used as positive controls, and solvents alone were used as negative controls and showed no zones of inhibition.

Given the partial reduction of berberine to **B10** gave promising improvement for a single structural modification, this was chosen as the path towards enhancing the activity of **B1**. As shown in Scheme 5, we reduced **B1** to produce **B14**, which was then screened against our panel of microbial organisms (Table 2). We were very pleased with the results of **B14**, representing a near universal improvement against all Gram-positive strains, with exceptional activity against *S. epidermidis*. This variant further enhanced the bacterial selectivity, as its heightened antibacterial levels were coupled with notable decreases in antifungal activity as compared to berberine. A graph showing a complete comparison of all variants and berberine is shown in Figure 3.

**Scheme 6:**
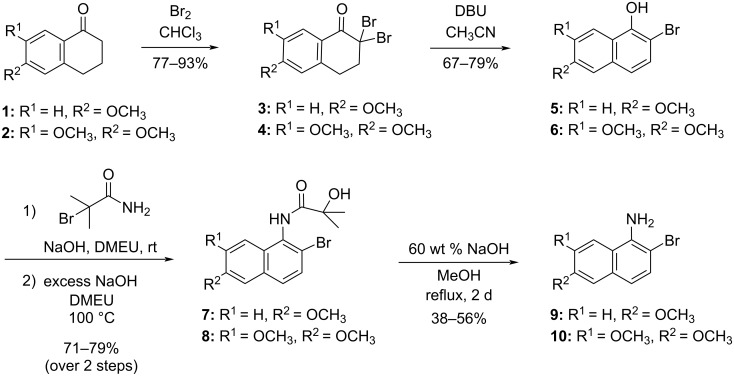
Synthesis of the substituted 2-bromoaminonaphthalenes **9** and **10**.

Having assessed the antimicrobial effects of the berberine variants, we then turned to the construction of chelerythrine variants. Numerous methods of synthesizing chelerythrine and other benzophenanthridines have been reported, but unlike with the berberine variants none are as easily modulated to rapidly install substituent diversity [31,43–48]. The method deemed most amenable to varying substituents involves substituted 2-bromo-1-aminonaphthalenes which are used in subsequent palladium cross-coupling reactions [45]. As such, our synthesis began with the generation of substituted 2-bromo-1-aminonaphthalenes **9** and **10** (Scheme 6). After α-bromination of tetralones **1** and **2**, intermediates **3** and **4** underwent elimination/aromatization with 1,8-diazabicyclo[5.4.0]undec-7-ene (DBU) to afford 2-bromo-1-naphthols **5** and **6** in fairly good yield. Conversion of naphthols to naphthylamines is typically achieved through a three-step sequence whereby the naphthol is first O-alkylated with 2-bromo-2-methylpropionamide and this ether undergoes a Smiles rearrangement to the hydroxyamide, which is hydrolyzed to the free naphthylamine [44–45]. It has previously been shown in the literature that the first two steps of this Smiles-rearrangement approach can be effectively performed in one pot [49]. Therefore, after allowing the O-alkylation to proceed at room temperature in dimethylethyleneurea (DMEU), we proceeded directly to the hydroxyamides **7** and **8** through the use of additional NaOH and refluxing the crude alkylation mixture. After heating for 3 h, the desired intermediates were recovered in acceptable yields over this two-step one-pot sequence. The amide hydrolysis of **7** and **8** to the desired free naphthylamines **9** and **10** proved challenging, but was nevertheless successful, albeit in low yields after an extended reaction time. We explored KOH and NH_3_ as alternatives to NaOH for this amide cleavage, and found the resulting yields less satisfactory. We also explored the amide hydrolysis under acidic conditions as well, though this resulted to almost complete decomposition.

**Scheme 7:**
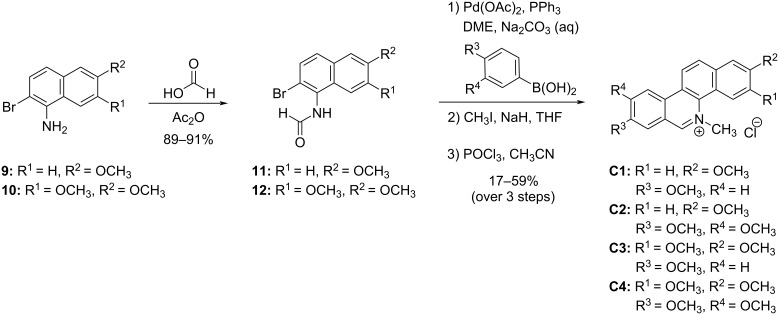
Completion of the synthesis of variants **C1**–**C4**.

With the desired naphthylamines in hand, we were able to complete our synthesis of four chelerythrine variants as shown in Scheme 7. After *N*-formylation providing intermediates **11** and **12** in good yield, a three-step sequence was performed: Suzuki coupling of the aryl bromide with one of two substituted arylboronic acids, followed by *N*-methylation, and final ring-closure via Bischler–Napieralski conditions [45,47–48]. These steps provided chelerythrine variants **C1**–**C4**, with structural variability stemming from the initial substituted tetralone (R^1^/R^2^) and/or the arylboronic acid (R^3^/R^4^). Compound **C4** is the known compound *O-*methylfagaronine, which has previously been synthesized through a variety of methods [50–52]. The antileukemia activity, antitumor activity, and inhibition of reverse transcriptase of *O-*methylfagaronine (**C4**) have previously been explored [51–54]. Despite this prior biological evaluation, compound **C4** was synthesized for assessment against our full panel of microbial organisms.

**Figure 4:**
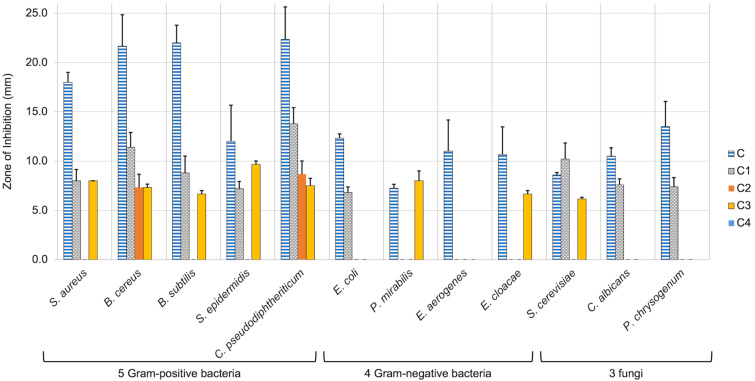
Kirby–Bauer zones of inhibition for variants **C1**–**C4** compared to original chelerythrine (**C**). Mean zones of inhibition using 0.12 mg of each compound tested against 12 unique microbial species shown with the associated standard error (*n* = 3–6). Vancomycin, streptomycin, and/or fluconazole were used as positive controls, and solvents were used as negative controls and showed no zones of inhibition.

The four chelerythrine variants were tested against our panel of microbes and the antimicrobial effects were compared to original chelerythrine (Table 3 and Figure 4). Given the greater synthetic effort needed for these variants, the results were overall disappointing. Only compounds **C1** and **C3** showed broad activity, and almost all variants were markedly less potent than original chelerythrine. The improved activity of the derivative **C1** against *S. cerevisiae* was the only notable exception.

**Table 3:** Zones of inhibition of chelerythrine variants **C1**–**C4** compared to chelerythrine (**C**).

Microbe	Mean zones of inhibition (mm)^a^				
	**C** (*n* = 6)	**C1** (*n* = 5)	**C2** (*n* = 3)	**C3** (*n* = 3)	**C4** (*n* = 3)

*S. aureus*	18.0	8.0	–	8.0	–
*B. cereus*	21.7	11.4	7.3	7.3	–
*B. subtilis*	22.0	8.8	–	6.7	–
*S. epidermidis*	12.0	7.2	–	9.7	–
*C. pseudodiphtheriticum*	22.3	13.8	8.7	7.5	–
*E. coli*	12.3	6.8	–	–	–
*P. mirabilis*	7.3	–	–	8.0	–
*E. aerogenes*	11.0	–	–	–	–
*E. cloacae*	10.7	–	–	6.7	–
*S. cerevisiae*	8.6	10.2	–	6.2	–
*C. albicans*	10.5	7.6	–	–	–
*P. chrysogenum*	13.5	7.4	–	–	–

^a^Mean zones of inhibition in millimeters for 0.12 mg of each compound. A dash (–) indicates no measurable antimicrobial effect. Vancomycin, streptomycin, and/or fluconazole were used as positive controls, and solvents were used as negative controls and showed no zones of inhibition.

Much of the disappointment in comparing the chelerythrine series stems from the high potency of chelerythrine itself. It should be noted that while the antibacterial effects of derivative **C1** are much lower than chelerythrine (**C**), all antibacterial results for the derivative **C1** are higher than that of berberine (**B**), and the effects of **C1** against *B. cereus* and *C. pseudodiphtheriticum* are stronger than almost all berberine variants. When looking at negative effects across all variants **B1**–**B14**; **C1**–**C4**, a trend was also observed with respect to a deleterious effect of the placement of a particular methoxy group. The ‘R^1^’ position within the generalized structure of the berberine variants (see Scheme 2) is analogous to the ‘R^4^’ position within the generalized chelerythrine variant structures (see Scheme 7). For all variants that only differed by the presence or absence of a methoxy group at this position, there was a decrease in activity for the variants bearing that methoxy substituent. Examples of note include compound **C2** being significantly weaker than **C1**, the complete inactivity of derivative **C4** compared to **C3**, **B8** being generally weaker than **B7**, and while **B5** still maintained decent activity, it was typically weaker than **B1**. The major exception to the deleterious effect of this methoxy group was the Gram-negative activity for **B5**, while **B1** lacked Gram-negative activity at the dosage investigated. A similar, though less universal trend was seen with the presence or absence of a methoxy group at position “R^5^” in the berberine series, which is analogous to position “R^2^” in the chelerythrine series. Generally speaking, **B7** showed a stark decrease compared to **B1**, **B8** was dramatically worse than **B5**, and **C3** often showed a diminished potency compared to **C1**. Perhaps the most noteworthy structure–activity relationship was one mentioned earlier, with the complete obliteration of activity seen with the 13-hydroxy-substituted berberine variants; though no analogous feature exists within the chelerythrine series.

Identifying the primary mechanism of action for these variants is complicated by both berberine and chelerythrine having multiple pathways associated with their antimicrobial activity [11–18,24]. In an effort to better understand the differences seen with our variants, we chose to focus on their effects on leakage of intercellular proteins, as disruption of cell wall permeability is associated with both parent phytochemicals. This antimicrobial effect has been studied using a bacterial alkaline phosphatase (ALP) assay to measure ALP leakage [11]. We compared the mean ALP activity in *S. aureus* for original berberine with ALP levels seen with a selection of berberine variants that were both more active (**B1**, **B3**, **B5**, and **B14**) and less active (**B2** and **B6**) (see Supporting Information File 1). While results varied between trials, it was found that variant **B14** did indeed result in an increase in measured extracellular ALP compared to berberine treatment, while the remaining variants resulted in either equal or lower concentrations of measured ALP levels (using a Mann–Whitney U Test and a significance cutoff of *P* ≤ 0.05). These results suggest intercellular protein leakage is not the primary mode of action for the improved activity seen in our variants, though it may be partially responsible for why variant **B14** was significantly more potent.

**Figure 5:**
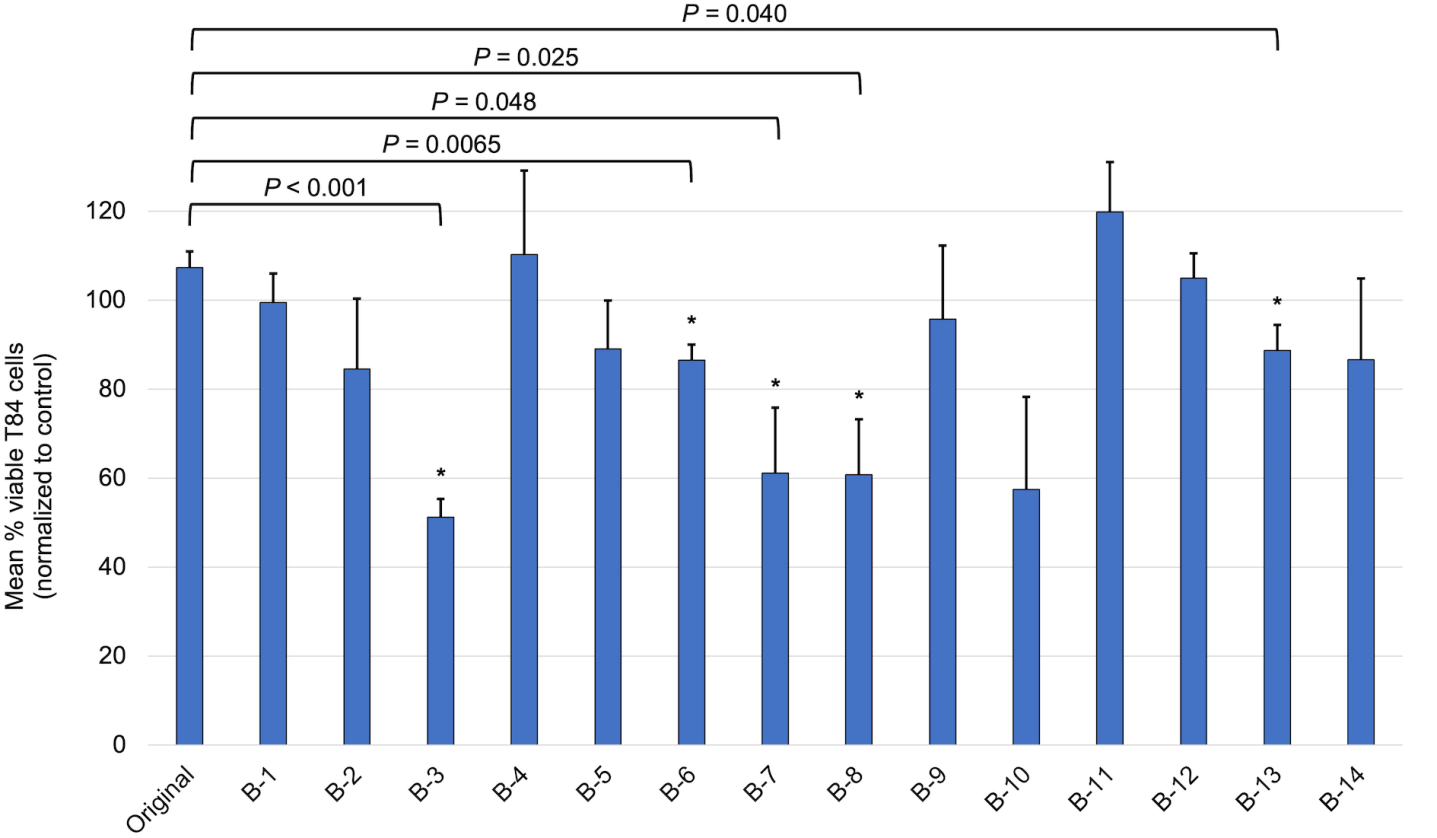
Effects of original berberine and all variants against T84 human colon cancer cells. Cells were treated for 1 h with 20 μL of a 6 mg/mL solution, resulting in treatments of 0.12 mg of the compound of interest. The MTT colorimetric assay was used to determine cell metabolic activity after treatment. The mean percentage of viable cells normalized to the control (solvent alone) is shown with associated standard error (*n* = 4). Significance was determined using two-tailed T-test analysis, with a significance cutoff of *P* ≤ 0.05. All significant differences are designated with an asterisk and the corresponding *P*-value displayed above.

Having preliminarily explored the antimicrobial activity of these variants, we next turned to investigating their effects on tumor cells, given our previous research had identified antitumor properties for the crude *A. mexicana* extract [9]. All variants were assessed against T84 colon cancer cells, using the MTT colorimetric assay, and compared to the parent compounds berberine or chelerythrine. As seen in Figure 5, several berberine variants showed fairly dynamic effects on the cancer cell viability, while original berberine was inactive at the dosage used (20 μL of a 6 mg/mL solution). A decrease in cell viability between 43–52% was seen for derivatives **B3**, **B7**, and **B8**, with all changes found to be of statistical significance (using two-tailed T-test analysis with significance set at *P* ≤ 0.05). There was also an average 46% decrease in viability for cells treated with compound **B10**, however, statistical analysis deemed this insignificant (*P* = 0.094). Given the low standard deviation for **B6** and **B13**, they did meet the criteria for statistical significance, although they only affected cell viability between 17–19%.

**Figure 6:**
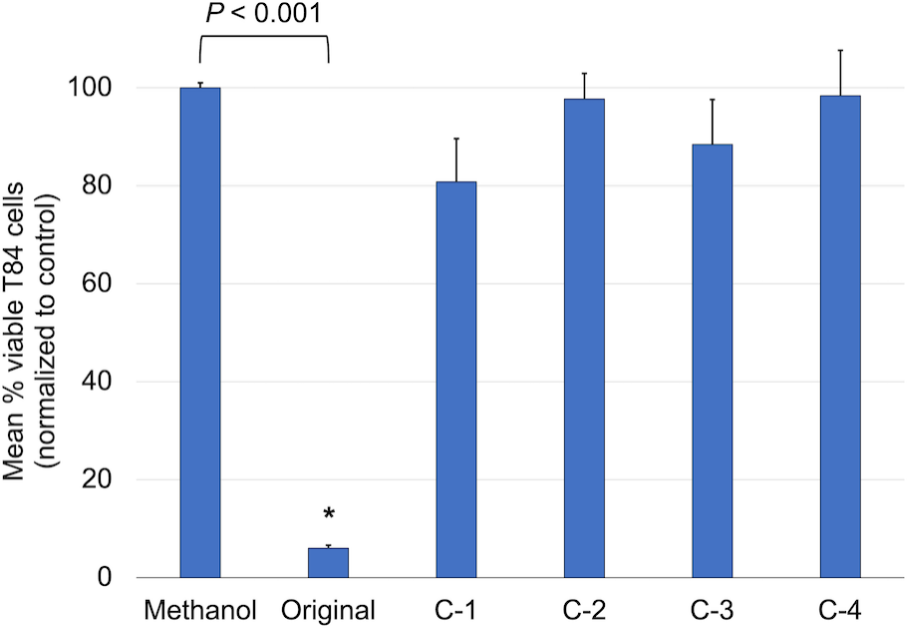
Effects of original chelerythrine and all variants against T84 human colon cancer. Cells were treated for 1 h with 5 μL of a 6 mg/mL solution, resulting in treatments of 0.03 mg of the compound of interest. The MTT colorimetric assay was used to determine cell metabolic activity after treatment. The mean percentage of viable cells normalized to the control (solvent alone) is shown with associated standard error (*n* = 4). Significance was determined using two-tailed T-test analysis, with a significance cutoff of *P* ≤ 0.05. Only original chelerythrine (*P* < 0.001) was determined to be significantly more potent than the methanol negative control.

Due to the high potency of chelerythrine against cancer cells, the dose for this series was lowered to 5 μL rather than the 20 μL used in the berberine series. Additionally, activity of the variants at this dosage was simply compared against the methanol blank (Figure 6). Similar to the antibacterial activity, the structural changes in the chelerythrine variants had a dramatically negative impact when compared to the parent structure. Slight activity was observed for compound **C1**, though at this low dosage it was still deemed statistically insignificant compared to the blank.

Screening these 20 unique compounds at a set concentration against T84 human colon cancer cells has provided useful preliminary comparative cytotoxicity data, which can be directly compared to our previous publication showing the effects of crude *A. mexicana* extracts against this same cell line [9]. However, further analyses utilizing a combination of methods could provide a better understanding of what happens in eukaryotic human cells after treatment with these plant-derived compounds. Future studies can include GI50 values to determine drug sensitivity for each compound in various cancerous vs non-cancerous human cell lines.

## Conclusion

Motivated by our prior isolation of three phytochemicals from the extracts of the *Argemone mexicana* plant, a library of structural variants of berberine and chelerythrine were prepared. Due to a greater synthetic ease, a larger number of berberine derivatives were explored. The structures of two unexpected oxidized berberine variants were elucidated through X-ray crystallography. Overall, the berberine series showed much greater promise, with several variants displaying heightened antibacterial activity compared to original berberine. Meanwhile the chelerythrine variants were notably less potent than the parent structure. The berberine-based compounds were predominantly active against Gram-positive bacteria, though some showed Gram-negative effects. Additionally, the berberine variants showing the greatest enhancement in activity (**B1** and **B14**) were overall less active towards fungi, suggesting prokaryotic selectivity. Some trends related to structure–activity relationships were observed, pointing to generally deleterious effects when additional oxygen-containing functional groups were incorporated at various positions throughout the ring systems. Furthermore, a number of berberine variants displayed promising preliminary results for cytotoxic effects against tumor cells.

## Supporting Information

File 1 Synthetic procedures, characterization, methods for biological testing, and copies of NMR spectra of new compounds.
