# Supplementary material for: Synthesis and biological evaluation of Argemone mexicana-inspired antimicrobials
Source: Beilstein J Org Chem. 2023 Sep 29;19:1511–24. doi: 10.3762/bjoc.19.108 (PMC10548253; doi:10.3762/bjoc.19.108)
Supplement: File 1 — Synthetic procedures, characterization, methods for biological testing, and copies of NMR spectra of new compounds. [file Beilstein_J_Org_Chem-19-1511-s001.pdf]

BEILSTEIN JOURNAL OF ORGANIC CHEMISTRY

Supporting Information

for

Synthesis and biological evaluation of *Argemone mexicana*-inspired antimicrobials

Jessica Villegas, Bryce C. Ball, Katelyn M. Shouse, Caleb W. VanArragon, Ashley N. Wasserman, Hannah E. Bhakta, Allen G. Oliver, Danielle A. Orozco-Nunnelly and Jeffrey M. Pruet

Beilstein J. Org. Chem. **2023,** *19,* 1511–1524. doi:10.3762/bjoc.19.108

Synthetic procedures, characterization, methods for biological testing, and copies of NMR spectra of new compounds

License and Terms: This is a supporting information file under the terms of the Creative Commons Attribution License (https://creativecommons.org/licenses/by/4.0). Please note that the reuse, redistribution and reproduction in particular requires that the author(s) and source are credited and that individual graphics may be subject to special legal provisions.
The license is subject to the *Beilstein Journal of Organic Chemistry* terms and conditions: (https://www.beilstein-journals.org/bjoc/terms)

Table of contents

- Methods S2
  - Synthesis S2–S12
  - X-Ray crystallography data S12–S14
  - Proposed mechanism for oxidative byproducts S14
  - Antimicrobial disc diffusion assay S15–S18
  - Alkaline phosphatase analysis S18–S19
  - Cell culture S20
  - Variant treatments and viability assay of colon cancer cells S20
- Copies of ^{1}H and ^{13}C spectra of new compounds S21–S35
- References S36

Methods:

Synthesis

General information: All reagents and solvents used were of commercial quality and obtained from Aldrich Chemical Co. and used as received. ^{1}H and ^{13}C NMR spectra were recorded in either DMSO-d_6, chloroform-d, D_2O, or methanol-d_4, with a Bruker spectrometer using the solvent as the reference. Chemical shifts are given in parts per million (ppm). Filtrations were performed by aid of reduced pressure. Evaporation of solvents was performed using a Büchi RE 121 rotary evaporator. Chromatographic purifications were performed on a CombiFlash Rf+ automated chromatography system (Teledyne Isco). Purity was assessed on a Waters UPLC with a QDa detector. High resolution mass spectrometry was performed with a Bruker micrOTOF-Q II. Berberine chloride (**B**), chelerythrine chloride (**C**), and 6-methoxy-1-tetralone (**1**) were purchased from Aldrich Chemical Co. and used as received. Compounds **B9** [1], **B10** [2], **B11** [2], **2** [3], **4** [4], **6** [4], **8** [4], **10** [4], and **12** [4] were all synthesized according to their literature procedures, and NMR data were in agreement with those reported [1-4].

Assignment of ^{1}H NMR signals was based on the numbering systems for berberine and chelerythrine shown in Figure S1 below [5,6].

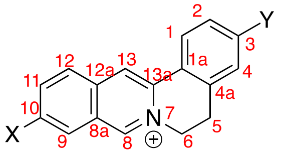

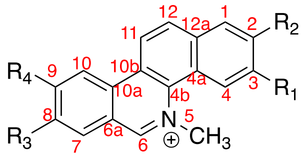

Figure S1: Numbering system for berberine and chelerythrine variants.

3,10-Dimethoxy-5,6-dihydroisoquinolino[3,2-*a*]isoquinolin-7-ium chloride (B1): To a 100 mL round-bottomed flask was added 1.22 mL (10 mmol) *m*-anisaldehyde and 30 mL of dichloromethane (DCM). To this was added 1.46 mL (10 mmol) 3-methoxyphenethylamine, and the reaction mixture was refluxed for 90 min with vigorous stirring. The reaction mixture was then cooled to room temperature, and the solvent was removed under vacuum. The crude oil was redissolved in 30 mL of methanol, and cooled to 0 °C with an ice bath for 5 min. To this was added 761 mg (20 mmol) $NaBH_4$ portion-wise over 5 min. The reaction mixture was allowed to come to room temperature, and stirred for an additional 2 h. After this time, the solvent was removed under

vacuum and the residue was treated with 50 mL of ethyl acetate and 20 mL of 0.1 M aqueous sodium carbonate. The layers were separated with a separatory funnel, and the organic layer was rinsed twice with brine. The organic layer was then dried with anhydrous sodium sulfate, filtered, and the solvent removed under vacuum. To the remaining oil was added 30 mL of formic acid, 2.9 g of a 40% (w/w) glyoxal solution (20 mmol glyoxal), and 3.2 g (20 mmol) anhydrous $CuSO_4$. The reaction mixture was stirred at 80 °C for 4 h, then cooled, treated with a large excess of CaO, filtered, and the formic acid removed under vacuum. The orange slurry was treated with 5 mL of a 3 M methanolic HCl solution and cooled in an ice bath, producing a bright yellow solid. The solid was isolated by filtration, and rinsed with a small amount of ice-cold methanol. After drying in a vacuum desiccator, 2.03 g (62%) of **B1** was recovered. MP = 215 °C (dec.) ^{1}H NMR (400 MHz, DMSO-*d*$_6$) δ(ppm) = 9.76 (s; 1H, H-8), 8.97 (s; 1H, H-13), 8.21 (d, *J* = 9.2Hz; 1H, H-12), 8.16 (d, *J* = 8.4Hz; 1H, H-11), 7.86-7.83 (dd, *J* = 9.2Hz, 2.4Hz; 1H, H-1), 7.74 (d, = 1.6Hz; 1H, H-9), 7.15-7.12 (dd, *J* = 8.8Hz, 2.4Hz; 1H, H-2), 7.10 (d, *J* = 2Hz; 1H, H-4), 4.89 (t, *J* = 6Hz; 2H, H-6), 4.01 (s; 3H, OCH_3), 3.89 (s; 3H, OCH_3), 3.29 (t, *J* = 6.4Hz; 2H, H-5). ^{13}C NMR (100 MHz, DMSO-*d*$_6$) δ(ppm) = 162.1, 160.4, 148.1, 138.8, 137.5, 135.0, 130.2, 129.5, 128.2, 127.8, 120.5, 120.1, 114.9, 113.7, 106.8, 56.6, 56.1, 55.6, 27.1. HRMS-ESI (*m/z*) [M]+ calc. for $(C_{19}H_{18}NO_2)^+$ 292.1332; found 292.1332.

11-Fluoro-13-hydroxy-3,10-dimethoxy-5,6-dihydroisoquinolino[3,2-*a*]isoquinolin-7-ium chloride (B2): This compound was prepared through an analogous procedure as described for **B1**, starting with 1.54 g (10 mmol) 4-fluoro-3-methoxybenzaldehyde, 1.46 mL (10 mmol) 3-methoxyphenethylamine, and 760 mg (20 mmol) $NaBH_4$. After cyclization with glyoxal as described previously, precipitation of the yellow product with methanolic HCl, and drying under vacuum, 1.92 g (53%) of **B2** was recovered. MP = 223 °C (dec.). ^{1}HNMR (400MHz, DMSO-*d*$_6$) δ (ppm) = 8.90 (d, *J* = 9.6Hz; 1H, H-8), 8.66 (s; 1H, H-9), 8.17 (d, *J* = 12Hz; 1H, H-12), 7.70 (d, *J* = 8.4Hz; 1H, H-1), 6.97 (s; 1H, H-4), 6.96 (d, *J* = 4.4Hz; 1H, H-2), 4.67 (t, *J* = 6Hz; 2H, H-6), 4.02 (s; 3H, OCH_3), 3.84 (s; 3H, OCH_3), 3.15 (t, *J*=6Hz; 2H, H-5). ^{13}C NMR (100 MHz, DMSO-*d*$_6$) δ(ppm) = 164.4, 158.3, 155.5, 152.9, 149.9, 135.3, 130.8, 129.2, 125.9, 123.3, 121.2, 112.7, 111.9, 110.1, 108.4, 56.7, 56.4, 55.6, 28.4. HRMS-ESI (*m/z*) [M]+ calc. for $(C_{19}H_{17}FNO_3)^+$ 326.1187; found 326.1177.

9-Fluoro-3,10-dimethoxy-5,6-dihydroisoquinolino[3,2-*a*]isoquinolin-7-ium chloride (B3) and **9-fluoro-13-hydroxy-3,10-dimethoxy-5,6-dihydroisoquinolino[3,2-*a*]isoquinolin-7-ium**

chloride (B4): This synthesis was carried out by an analogous method as described for **B1**, starting with 1.54 g (10 mmol) 2-fluoro-3-methoxybenzaldehyde, 1.46 mL (10 mmol) 3-methoxyphenethylamine, and 762 mg (20 mmol) $NaBH_4$. Cyclization with glyoxal as described previously and precipitation of the yellow product with methanolic HCl resulted in a roughly 1:1 ratio of **B3:B4**, as determined by NMR. The two products were chromatographically separated on a CombiFlash Rf+ using a pre-packed silica column, and initially eluting with a 95:5 DCM/MeOH mobile phase, followed by a 9:1 DCM/MeOH (0.5% NH_3) mobile phase. Pooling and evaporating fractions containing the second column component provided 1.07 g (31%) of **B3** after drying under vacuum. MP = 286 °C (dec.). ^{1}HNMR (400MHz, CD_3OD) δ (ppm) = 9.85 (s; 1H, H-8), 8.84 (s; 1H, H-13), 8.19-8.10 (m; 3H, H-11 & H-12 & H-1), 7.13-7.10 (dd, J = 6Hz, 2.8Hz; 1H, H-2), 7.05 (d, J = 2.4Hz; 1H, H-4), 4.97 (t, J = 6.4Hz; 2H, H-6), 4.16 (s; 3H, OCH_3), 3.92 (s; 3H, OCH_3), 3.35 (t, J = 6.4Hz; 2H, H-5). ^{13}C NMR (100 MHz, CD_3OD) δ(ppm) = 162.8, 147.4, 146.1, 143.1, 139.5, 137.0, 133.4, 127.7, 127.5, 124.3, 119.9, 119.2, 117.9, 114.5, 112.9, 56.9, 55.9, 54.8, 26.9. HRMS-ESI (*m/z*) [M]+ calc. for $(C_{19}H_{17}FNO_2)^+$ 310.1238; found 310.1245.

Pooling and evaporating fractions containing the first column component provided 1.18 g (33%) of **B4** after drying under vacuum. MP = 204 °C (dec.). ^{1}HNMR (400MHz, CD_3OD) δ (ppm) = 9.42 (s; 1H, H-8), 8.52 (d, J = 8.8Hz; 1H, H-12), 8.33 (d, J = 9.6Hz; 1H, H-1), 8.11 (t, J = 8.4Hz; 1H, H-11), 7.06 (d, J = 2.8Hz; 1H, H-2), 7.03 (s; 1H, H-4), 4.86 (t, J = 6Hz; 2H, H-6), 4.15 (s; 3H, OCH_3), 3.91 (s; 3H, OCH_3), 3.26 (t, J = 6Hz; 2H, H-5). ^{13}C NMR (100 MHz, DMSO-d_6) δ(ppm) = 160.5, 151.6, 146.0, 144.6, 138.4, 132.7, 131.4, 126.9, 126.0, 124.3, 121.3, 119.6, 118.3, 113.3, 113.1, 57.8, 57.0, 55.9, 27.8. HRMS-ESI (*m/z*) [M]+ calc. for $(C_{19}H_{17}FNO_3)^+$ 326.1187; found 326.1184.

Optimized conditions for the synthesis of 9-fluoro-3,10-dimethoxy-5,6-dihydroisoquinolino[3,2-*a*]isoquinolin-7-ium chloride (B3): The reductive amination to the secondary amine was achieved exactly as before, using 1.54 g (10 mmol) 2-fluoro-3-methoxybenzaldehyde, 1.46 mL (10 mmol) 3-methoxyphenethylamine, and 760 mg (20 mmol) $NaBH_4$. However, after the crude oil was dissolved in 30 mL formic acid, it was first heated to 80 °C with the 2.9 g of a 40% (w/w) glyoxal solution (20 mmol glyoxal) for 90 min, to initiate cyclization prior to the addition of $CuSO_4$. After the initial 90 min of heating, 1.6 g (10 mmol) anhydrous $CuSO_4$ was added, and the reaction continued with stirring at 80 °C for an additional 2.5 h. Work-up proceeded as before, with addition of excess CaO, filtration, removal of formic

acid under vacuum, and treatment with cold methanolic HCl. This resulted in almost exclusively **B3**, which was further purified on a CombiFlash Rf+ eluting with a 9:1 DCM/MeOH (0.5% NH_3) mobile phase. After pooling and evaporating fractions, 2.04 g (59%) of **B3** was isolated and was spectroscopically identical to that prepared as described above.

3,10,11-Trimethoxy-5,6-dihydroisoquinolino[3,2-*a*]isoquinolin-7-ium chloride (B5) and **13-hydroxy-3,10,11-trimethoxy-5,6-dihydroisoquinolino[3,2-*a*]isoquinolin-7-ium chloride (B6):** This synthesis was carried out by an analogous method as described for **B1**, starting with 831 mg (5 mmol) 3,4-dimethoxybenzaldehyde, 0.73 mL (5 mmol) 3-methoxyphenethylamine, and 380 mg (10 mmol) $NaBH_4$. Cyclization with glyoxal as described previously and precipitation of the yellow product with methanolic HCl resulted in a roughly 9:1 ratio of **B5/B6**, as determined by NMR. The two products were chromatographically separated on a CombiFlash Rf+ using a pre-packed silica column, and initially eluting with 95:5 DCM/MeOH mobile phase, followed by a 9:1 DCM/MeOH (0.5% NH_3) mobile phase. Pooling and evaporating fractions containing the second column component provided 1.10 g (62%) of **B5** after drying under vacuum. MP = 212 °C (dec.). The collected spectral data was consistent with that reported from an alternative synthesis of this compound [7].

Pooling and evaporating fractions containing the first column component provided 130 mg (7%) of **B6** after drying under vacuum. MP = 232 °C (dec.). ^{1}HNMR (400MHz, CD_3OD) δ (ppm) = 9.08 (d, J = 8.8Hz; 1H, H-8), 8.19 (s; 1H, H-12), 7.82 (s; 1H, H-1), 7.28 (s; 1H, H-9), 6.94-6.92 (dd, J = 6Hz, 2.8Hz; 1H, H-2), 6.87 (d, J = 2.8Hz; 1H, H-4), 4.59 (t, J = 6Hz; 2H, H-6), 4.04 (s; 3H, OCH_3), 3.98 (s; 3H, OCH_3), 3.85 (s; 3H, OCH_3), 3.14 (t, J = 6.4Hz; 2H, H-5). ^{13}C NMR (100 MHz, CD_3OD) δ(ppm) = 161.3, 159.2, 154.2, 152.2, 135.7, 132.1, 129.7, 126.5, 124.9, 123.2, 122.1, 112.1, 111.6, 105.1, 102.5, 56.2, 55.2, 55.1, 54.4, 28.4. HRMS-ESI (*m/z*) [M]+ calc. for $(C_{20}H_{20}NO_4)^+$ 338.1387; found 338.1391.

2,3,10-Trimethoxy-5,6-dihydroisoquinolino[3,2-*a*]isoquinolin-7-ium chloride (B7): This compound was synthesized in an analogous way to the optimized method described for **B3**, starting with 0.36 mL (3 mmol) *m*-anisaldehyde, 0.51 mL (3 mmol) 3,4-dimethoxyphenethylamine, and 227 mg (6 mmol) $NaBH_4$. The secondary amine was heated to 80 °C in 10 mL formic acid with 870 mg of 40% (w/w) glyoxal solution (6 mmol glyoxal) for 90 min, and then 479 mg (3 mmol) $CuSO_4$ was added and the reaction maintained at 80 °C for an additional 2.5 h. Work-up proceeded as before, with addition of excess CaO, filtration, removal of formic acid under vacuum, and

treatment with cold methanolic HCl. After filtration and drying under vacuum, 612 mg (57%) of **B7** was isolated. MP = 280 °C (dec.). ^{1}HNMR (400MHz, DMSO-d_6) δ (ppm) = 9.77 (s; 1H, H-8), 9.04 (s; 1H, H-13), 8.20 (d, *J* = 9.2Hz; 1H, H-12), 7.88-7.85 (dd, *J* = 6.4Hz, 2.4Hz; 1H, H-11), 7.76 (d, *J* = 2.4Hz; 1H, H-9), 7.73 (s; 1H, H-1), 7.12 (s; 1H, H-4), 4.87 (t, *J* = 6Hz; 2H, H-6), 4.01 (s; 3H, OCH_3), 3.95 (s; 3H, OCH_3), 3.88 (s; 3H, OCH_3), 3.25 (t, *J* = 6.4Hz; 2H, H-5). ^{13}C NMR (100 MHz, DMSO-d_6) δ(ppm) = 160.4, 152.1, 149.2, 148.3, 138.8, 134.9, 130.3, 129.4, 129.0, 127.7, 120.6, 119.4, 111.8, 109.3, 106.9, 56.7, 56.6, 56.3, 55.9, 26.4. HRMS-ESI (*m/z*) [M]+ calc. for $(C_{20}H_{20}NO_3)^+$ 322.1438; found 322.1431.

2,3,10,11-Tetramethoxy-5,6-dihydroisoquinolino[3,2-*a*]isoquinolin-7-ium chloride (B8): This compound was synthesized in an analogous way to the optimized method described for **B3** and **B7**, starting with 332 mg (2 mmol) 3,4-dimethoxybenzaldehyde, 0.34 mL (2 mmol) 3,4-dimethoxyphenethylamine, and 152 mg (4 mmol) $NaBH_4$. The secondary amine was heated to 80 °C in 10 mL formic acid with 580 mg of 40% (w/w) glyoxal solution (4 mmol glyoxal) for 90 min, and then 320 mg (2 mmol) $CuSO_4$ was added and the reaction maintained at 80 °C for an additional 2.5 h. Work-up proceeded as before, with addition of excess CaO, filtration, removal of formic acid under vacuum, and treatment with cold methanolic HCl. After filtration and drying under vacuum, 395 mg (51%) of **B8** was isolated. MP = 221 °C (dec.). (lit. 220-222 °C) [8]. The collected spectral data was consistent with that reported in an alternative synthesis of the compound [7].

1-(9,10-Dimethoxy-5,8-dihydro-6*H*-[1,3]dioxolo[4,5-*g*]isoquinolino[3,2-*a*]isoquinolin-8-yl)propan-2-one (B9): This compound was synthesized according to its literature procedure, and the spectral data was in agreement with those reported [1].

9,10-Dimethoxy-5,8-dihydro-6*H*-[1,3]dioxolo[4,5-*g*]isoquinolino[3,2-*a*]isoquinoline (B10): This compound was synthesized according to its literature procedure, and the spectral data was in agreement with those reported [2].

9,10-Dimethoxy-5,8,13,13a-tetrahydro-6*H*-[1,3]dioxolo[4,5-*g*]isoquinolino[3,2-*a*]isoquinoline (B11): This compound was synthesized according to its literature procedure, and the spectral data was in agreement with those reported [2].

***N*-(3-Methoxybenzyl)-2-(3-methoxyphenyl)-*N*,*N*-dimethylethan-1-aminium iodide (B12):** The initial reductive amination step was performed exactly as described for the first stage of preparation of **B1**, using 1.22 mL (10 mmol) *m*-anisaldehyde, 1.45 mL (10 mmol) 3-

methoxyphenethylamine, and 760 mg (20 mmol) $NaBH_4$. The crude oil of the secondary amine was dissolved in 30 mL acetonitrile, followed by addition of 2.7 g (20 mmol) K_2CO_3 and 6 mL (96 mmol) iodomethane. After stirring at 50 °C for 5 h the reaction mixture was concentrated under vacuum and then suspended in 20 mL of ice-cold diethyl ether and filtered. The isolated solid was dissolved in a 9:1 mixture of $CHCl_3$/MeOH and the insoluble K_2CO_3 was removed by filtration. Evaporation of the filtrate under vacuum provided 4.14 g (97%) of the quaternary ammonium compound **B12**. MP = 176 °C. ^{1}HNMR (400MHz, D_2O) δ (ppm) = 7.51 (t, J = 7.6Hz; 1H, Ar-H), 7.38 (t, J = 7.6Hz; 1H, Ar-H), 7.21 (d, J = 8.4Hz; 2H, Ar-H), 7.14 (s; 1H, Ar-H), 6.99-6.95 (m; 3H, Ar-H), 4.56 (s; 2H, Ar-CH_2-N), 3.86 (s; 3H, OCH_3), 3.85 (s; 3H, OCH_3), 3.57-3.53 (m; 2H, N-CH_2), 3.26-3.21 (m; 2H, Ar-CH_2), 3.14 (s; 6H, 2x N^+-CH_3). ^{13}C NMR (100 MHz, DMSO-d_6) δ(ppm) = 159.9, 159.8, 138.3, 130.5, 130.2, 129.8, 125.5, 121.6, 119.1, 116.2, 115.2, 113.0, 66.6, 64.6, 55.9, 55.7, 49.9, 28.7. HRMS-ESI (*m/z*) [M]+ calc. for $(C_{19}H_{26}NO_2)^+$ 300.1958; found 300.1959.

***N*-(3-Methoxybenzyl)-2-(3-methoxyphenyl)ethan-1-aminium chloride (B13):** The initial reductive amination step was performed exactly as described for **B1** and **B12**, using 1.22 mL (10 mmol) *m*-anisaldehyde, 1.45 mL (10 mmol) 3-methoxyphenethylamine, and 760 mg (20 mmol) $NaBH_4$. The crude oil of the secondary amine was dissolved in 15 mL of ethyl acetate and after cooling in an ice-bath the solution was treated dropwise with 12 M HCl, resulting in a thick suspension. The solid was isolated by filtration and rinsed with 20 mL ice-cold diethyl ether. Drying the solid in a vacuum desiccator provided 2.83 g (92%) of **B13**. MP = 131 °C. ^{1}HNMR (400MHz, D_2O) δ (ppm) = 7.45 (t, J = 8Hz; 1H, Ar-H), 7.37 (t, J = 8Hz; 1H, Ar-H), 7.12-7.06 (m; 2H, Ar-H), 7.03 (t, J = 2.1Hz; 1H, Ar-H), 6.97-6.93 (td, J = 7.4Hz, 2.6Hz; 2H, Ar-H), 6.88 (t, J = 1.9Hz; 1H, Ar-H), 4.23 (s; 2H, Ar-CH_2-N), 3.86 (s; 3H, OCH_3), 3.84 (s; 3H, OCH_3), 3.34 (t, J = 7.6Hz; 2H, N-CH_2), 3.03 (t, J = 7.6Hz; 2H, Ar-CH_2). ^{13}C NMR (100 MHz, D_2O) δ(ppm) = 159.3, 159.2, 138.2, 132.1, 130.6, 130.3, 122.4, 121.5, 115.2, 115.1, 114.4, 112.9, 55.4, 55.3, 50.7, 47.5, 31.5. HRMS-ESI (*m/z*) [M]+ calc. for $(C_{17}H_{22}NO_2)^+$ 272.1645; found 272.1644.

3,10-Dimethoxy-5,8-dihydro-6*H*-isoquinolino[3,2-*a*]isoquinoline (B14): To a 25 mL round-bottomed flask was added 370 mg (1.13 mmol) **B1** and 414 mg (3 mmol) K_2CO_3, followed by 14 mL methanol, and the solution was stirred at room temperature. Separately, 46 mg (1.25 mmol) $NaBH_4$ was dissolved in 0.5 mL of 1 M aqueous NaOH and this was added dropwise to the stirred solution of **B1** from above. The reaction was allowed to proceed for 30 min at room temperature,

and then the solid was collected by filtration. The precipitate was washed with 15 mL DI water, followed by 5 mL of methanol. After drying in a vacuum desiccator 200 mg (60%) of **B14** was isolated. MP = 143 °C (dec.). ^{1}HNMR (400MHz, $CDCl_3$) δ (ppm) = 7.66 (d, *J* = 8.8Hz; 1H, H-12), 6.95 (d, *J* = 8.4Hz; 1H, H-1), 6.80-6.77 (dd, *J* = 6Hz, 2.8Hz; 1H, H-11), 6.74-6.71 (dd, *J* = 6Hz, 2.4Hz; 1H, H-1), 6.64-6.62 (m; 2H, H-9 & H-4), 6.08 (s; 1H, H-13), 4.21 (s; 2H, Ar-CH_2-N), 3.82 (s; 3H, OCH_3), 3.79 (s; 3H, OCH_3), 3.14 (t, *J* = 5.6Hz; 2H, H-6), 2.95 (t, *J* = 5.6Hz; 2H, H-5). ^{13}C NMR (100 MHz, $CDCl_3$) δ(ppm) = 158.9, 157.4, 141.4, 135.6, 129.4, 127.8, 125.3, 124.2, 123.7, 113.0, 112.8, 112.4, 111.1, 96.1, 55.43, 55.38, 55.29, 49.0, 30.1. HRMS-ESI (*m/z*) [M+H]+ calc. for $(C_{19}H_{20}NO_2)^+$ 294.1489; found 294.1487.

6,7-Dimethoxy-3,4-dihydronaphthalen-1(2*H*)-one (2): This compound was synthesized according to its literature procedure, and spectral data was in agreement with those reported [3].

2,2-Dibromo-6-methoxy-3,4-dihydronaphthalen-1(2*H*)-one (3): A solution of 4.67 mL (91 mmol) of bromine in 20 mL of chloroform was added dropwise over the course of 1 h via an addition funnel to a solution of 7.6 g (43 mmol) of 6-methoxy-1-tetralone (**1**) in 40 mL of chloroform. The reaction was shielded from light with aluminum foil and left to stir overnight at room temperature. The reaction contents were transferred to a separatory funnel and rinsed three times with a 0.1 M aqueous $NaHCO_3$ solution. The organic layer was dried with anhydrous $MgSO_4$, filtered, and evaporation of the solvent yielded 13.36 g (93%) of **3**. MP = 84 °C (lit. 85 °C [9]). The collected NMR data was consistent with that reported from an alternative synthesis of this compound [9].

2,2-Dibromo-6,7-dimethoxy-3,4-dihydronaphthalen-1(2*H*)-one (4): This compound was synthesized according to its literature procedure, and the spectral data was in agreement with those reported [4].

2-Bromo-6-methoxynaphthalen-1-ol (5): A solution of 13 g (39 mmol) of **3** in 270 mL of acetonitrile was stirred for 15 min at 45 °C under N_2. Then, 8.9 mL (60 mmol) of 1,8-diazabicyclo[5.4.0]undec-7-ene (DBU) was added and the reaction mixture was left to stir for 1 h at 45 °C under N_2. The reaction was quenched with 200 mL of 1 M HCl and the product was extracted twice into dichloromethane (2 × 300 mL). The combined organic layer was dried with $MgSO_4$, filtered, and the solvent evaporated under vacuum. The crude product was purified via column chromatography (1:1 hexane/ethyl acetate) to provide 6.61 g (67%) of **5**. MP = 86 °C (lit.

86 °C [9]). The collected NMR data was consistent with that reported from an alternative synthesis of this compound [9].

2-Bromo-6,7-dimethoxynaphthalen-1-ol (6): This compound was synthesized according to its literature procedure, and the spectral data was in agreement with those reported [4].

***N*-(2-Bromo-6-methoxynaphthalen-1-yl)-2-hydroxy-2-methylpropanamide (7):** To a stirred solution of 6.5 g (26 mmol) of **5** in 45 mL of dimethylethyleneurea (DMEU) was added 5 g (125 mmol) of finely powdered NaOH, and the solution was stirred for 15 min at room temperature. To this was added 11.8 g (71 mmol) of 2-bromo-2-methylpropionamide and the mixture was stirred for 4 h at room temperature, at which point TLC showed complete disappearance of the starting material. An additional 1 g (25 mmol) of powdered NaOH was added and the reaction mixture was heated to 100 °C to initiate the Smiles rearrangement. After being maintained at 100 °C for 3 h the reaction mixture was cooled to room temperature and added to 150 mL of DI water, followed by addition of 25 mL of 6 M HCl to neutralize the excess NaOH. The suspension was maintained in an ice bath for 1 h, at which point the solid product was collected by filtration and washed several times with water. Drying the product overnight in a vacuum oven over P_2O_5 provided 6.24 g (71% over two steps) of **7**. MP = 221 °C. ^{1}HNMR (400MHz, $CDCl_3$) δ (ppm) = 8.48 (s; NH), 7.68 (d, *J* = 9.6Hz; 1H), 7.59 (d, *J* = 8.8Hz; 1H), 7.54 (d, *J* = 8.8Hz; 1H), 7.18-7.15 (dd, *J* = 6.8Hz, 2.4Hz; 1H), 7.09 (d, *J* = 2.4; 1H), 3.90 (s; 3H), 2.6-2.4 (br, OH), 1.67 (s; 6H). ^{13}C NMR (100 MHz, $CDCl_3$) δ(ppm) = 175.3, 158.0, 134.5, 131.2, 129.9, 127.7, 126.7, 125.2, 119.9, 117.0, 106.1, 74.4, 55.4, 28.0. HRMS-ESI (*m/z*) [M+H]+ calc. for $(C_{15}H_{17}BrNO_3)^+$ 338.0386; found 338.0385.

***N*-(2-Bromo-6,7-dimethoxynaphthalen-1-yl)-2-hydroxy-2-methylpropanamide (8):** This compound was synthesized according to its literature procedure, and the spectral data was in agreement with those reported [4].

2-Bromo-6-methoxynaphthalen-1-amine (9): To a solution of 2.5 g (7.4 mmol) of **7** in 25 mL of methanol was added 50 mL of 60 wt % aqueous NaOH and the reaction mixture was refluxed for 2 days. The mixture was added to 120 mL of DI water and cooled in an ice bath, and then extracted with ethyl acetate (4 × 150 mL). The pooled organic layers were dried with anhydrous Na_2SO_4, filtered, and the solvent removed under vacuum. The residue was purified by column chromatography (7:3 hexane/ethyl acetate) to provide 1.04 g (56%) of **9**. MP = 111 °C. ^{1}HNMR (400MHz, $CDCl_3$) δ (ppm) = 7.69 (d, *J* = 9.2Hz; 1H), 7.42 (d, *J* = 8.8Hz; 1H), 7.13-7.10 (dd, *J* =

9.2Hz, 2.4Hz; 1H), 7.07-7.05 (m; 2H), 4.62-4.44 (br, NH_2), 3.91 (s; 3H). ^{13}C NMR (100 MHz, $CDCl_3$) δ(ppm) = 157.8, 139.6, 134.7, 130.4, 122.6, 119.0, 118.4, 118.0, 106.8, 102.2, 55.3. HRMS-ESI (*m/z*) [M+H]+ calc. for $(C_{11}H_{11}BrNO)^+$ 252.0018; found 252.0020.

2-Bromo-6,7-dimethoxynaphthalen-1-amine (10): This compound was synthesized according to its literature procedure, and the spectral data was in agreement with those reported [4].

***N*-(2-Bromo-6-methoxynaphthalen-1-yl)formamide (11):** An acetic-formic anhydride was generated by dropwise addition of 0.5 mL (13 mmol) of formic acid to 0.95 mL (10 mmol) of acetic anhydride at 0 ºC. The reaction mixture was gently warmed to 50 °C and left to stir for 2 h. The reaction mixture was cooled to room temperature and diluted with 1 mL of anhydrous tetrahydrofuran (THF). A solution of 1.0 g (4 mmol) of **9** in 8 mL of THF was added to the reaction mixture, which was left to stir for 45 min. The volatiles were removed under vacuum and the remaining solid was suspended in 15 mL ethyl acetate, filtered, washed with cold ethyl acetate, and dried under vacuum to provide 1.0 g (89%) of **11**, which was observed as a 4:1 ratio of rotamers. MP = 220 °C (dec.). ^{1}HNMR (400MHz, DMSO-d_6) δ (ppm) = {two rotamers} [10] 10.13 (s; 1H) {10.05 minor rotamer}, 8.45 (s; NH) {8.15 minor rotamer}, 7.79 (s; 1H) {7.90 minor rotamer}, 7.77-7.75 (m, 1H) {overlapping minor rotamer}, 7.70 (d, J = 8.8Hz; 1H) {7.75 minor rotamer}, 7.41 (d, J = 2.4Hz; 1H) {7.42 minor rotamer}, 7.27-7.24 (dd, J = 9.2Hz, 2.4Hz; 1H) {7.3 minor rotamer}, 3.89 (s; 3H) {3.89 minor rotamer}. ^{13}C NMR (100 MHz, $CDCl_3$) δ(ppm) = 160.6, 158.2, 134.6, 132.1, 130.4, 128.2, 126.9, 125.9, 120.3, 117.5, 106.9, 55.9. HRMS-ESI (*m/z*) [M+H]+ calc. for $(C_{12}H_{11}BrNO_2)^+$ 279.9968; found 279.9967.

***N*-(2-Bromo-6,7-dimethoxynaphthalen-1-yl)formamide (12):** This compound was synthesized according to its literature procedure, and spectral data was in agreement with those reported [4].

2,8-Dimethoxy-5-methylbenzo[*c*]phenanthridin-5-ium chloride (C1): The compound was synthesized over a three-step sequence. Initially, 490 mg (1.75 mmol) of **11** was dissolved in 8 mL of 1,2-dimethoxyethane and flushed with argon. To this was added 12 mg (0.05 mmol) of palladium(II) acetate and 27 mg (0.1 mmol) of triphenylphosphine, and the reaction mixture was flushed again with argon before stirring for 10 min. Then, 2.6 mL of 2 M Na_2CO_3 was added to the reaction mixture, and it was again flushed with argon before stirring for one hour. To the mixture was added 425 mg (2.8 mmol) of 4-methoxyphenylboronic acid and stirring was continued for 3 h. The reaction mixture was diluted with 20 mL of ethyl acetate, added to a separatory funnel, and washed with 10 mL of DI water. The organic layer was then filtered through a short plug of

SiO_2, which was further eluted with additional ethyl acetate. The pooled organic filtrate was concentrated under vacuum to provide the crude Suzuki-coupled intermediate, which was immediately re-dissolved in 20 mL anhydrous THF, cooled to 0 °C in an ice bath, and treated with 72 mg sodium hydride (60% dispersion in mineral oil) under N_2. The reaction mixutre was stirred for 20 min, and allowed to come to room temperature, before being treated with 125 μL (2 mmol) iodomethane. This reaction mixture was stirred for 1 h before being added to 30 mL of 0.25 M HCl and extracted with dichloromethane (2 × 100 mL). The combined organic layers were dried with anhydrous $MgSO_4$, filtered, and the solvent removed under vacuum to give a crude methylated formamide. This crude material was then submitted to Bischler–Napieralski cyclization by dissolving the residue in 20 mL acetonitrile and treating with 2 mL phosphorous oxychloride. This reaction mixture was refluxed for 2 h, at which time it was allowed to cool and then poured into a 30 mL mixture of 0.1 M HCl and crushed ice. A bright yellow solid precipitated upon standing for 5 min. The solid was isolated by vacuum filtration, and rinsed with ice cold water. Chromatographic purification (95:5 DCM/MeOH) provided 101 mg (17% over three steps) of **C1**. MP = 248 °C (dec.). ^{1}HNMR (400MHz, CD_3OD) δ (ppm) = 9.92 (s; 1H, H-6), 9.00 (d, J = 9.2Hz; 1H, H-10), 8.88 (d, J = 9.6Hz; 1H, H-11), 8.83 (d, J = 9.2Hz; 1H, H-4), 8.35 (d, J = 9.2Hz; 1H, H-12), 8.00-7.98 (dd, J = 6.8Hz, 2.4Hz; 1H, H-9), 7.89 (d, J = 2.8Hz; 1H, H-7), 7.72 (d, J = 2.8Hz; 1H, H-1), 7.55-7.52 (dd, J = 6.4Hz, 2.8Hz; 1H, H-3), 5.08 (s; 3H, N^+-CH_3), 4.11 (s; 3H, OCH_3), 4.06 (s; 3H, OCH_3). ^{13}C NMR (100 MHz, CD_3OD) δ(ppm) = 160.6, 159.9, 152.6, 137.3, 133.1, 131.5, 130.1, 129.8, 128.0, 125.8, 125.3, 124.7, 120.0, 118.5, 118.2, 108.8, 108.0, 55.4, 54.9, 51.8. HRMS-ESI (*m/z*) [M]+ calc. for $(C_{20}H_{18}NO_2)^+$ 304.1332; found 304.1330.

2,8,9-Trimethoxy-5-methylbenzo[*c*]phenanthridin-5-ium chloride (C2): This compound was synthesized in an identical manner as that reported for **C1**, at the same scale, but the boronic acid was replaced with 509 mg (2.8 mmol) 3,4-dimethoxyphenylboronic acid. After completion of the three-step reaction sequence, precipitation from ice-cold dilute HCl, and chromatographic purification (95:5 DCM/MeOH), 382 mg (59% over three steps) of **C2** was isolated. MP = 245 °C (dec.). ^{1}HNMR (400MHz, CD_3OD) δ (ppm) = 9.68 (s; 1H, H-6), 8.89-8.86 (m; 2H, H-11 & H-4), 8.33-8.31 (m; 2H, H-10 & H-12), 7.84 (s; 1H, H-7), 7.72 (d, J = 2.8Hz; 1H, H-1), 7.55-7.52 (dd, J = 6.8Hz, 2.8Hz; 1H, H-3), 5.01 (s; 3H, N^+-CH_3), 4.29 (s; 3H, OCH_3), 4.14 (s; 3H, OCH_3), 4.08 (s; 3H, OCH_3). ^{13}C NMR (100 MHz, CD_3OD) δ(ppm) = 159.7, 159.1, 152.3, 149.9, 137.2, 133.1,

132.6, 130.4, 128.0, 123.6, 120.3, 119.9, 118.1, 117.9, 108.6, 107.8, 102.0, 56.4, 55.6, 54.9, 51.1. HRMS-ESI (*m/z*) [M]+ calc. for $(C_{21}H_{20}NO_3)^+$ 334.1438; found 334.1437.

2,3,8-Trimethoxy-5-methylbenzo[*c*]phenanthridin-5-ium chloride (C3): This compound was synthesized in an identical manner as that reported for **C1**, at the same scale, but compound **11** was replaced with 542 mg (1.75 mmol) of compound **12**. After completion of the three-step reaction sequence, precipitation from ice-cold dilute HCl, and chromatographic purification (95:5 DCM/MeOH), 168 mg (26% over three steps) of **C3** was isolated. MP = 221 °C (dec.). ^{1}HNMR (400MHz, CD_3OD) δ (ppm) = 9.91 (s; 1H, H-6), 8.91 (d, *J* = 9.2Hz; 1H, H-10), 8.66 (d, *J* = 8.8Hz; 1H, H-11), 8.27 (d, *J* = 8.8Hz; 1H, H-12), 8.16 (s; 1H, H-4) 7.95-7.92 (dd, *J* = 6.8Hz, 2.8Hz; 1H, H-9), 7.88 (d, *J* = 2.8Hz; 1H, H-7), 7.65 (s; 1H, H-1), 5.13 (s; 3H, N^+-CH_3), 4.17 (s; 3H, OCH_3), 4.11 (s; 3H, OCH_3), 4.09 (s; 3H, OCH_3). ^{13}C NMR (100 MHz, CD_3OD) δ(ppm) = 160.6, 152.5 151.3, 150.0, 132.0, 131.4, 130.9, 130.1, 129.6, 125.7, 125.6, 124.7, 118.8, 117.7, 108.5, 108.2, 107.2, 55.5, 55.4, 55.2, 51.5. HRMS-ESI (*m/z*) [M]+ calc. for $(C_{21}H_{20}NO_3)^+$ 334.1438; found 334.1439.

2,3,8,9-Tetramethoxy-5-methylbenzo[*c*]phenanthridin-5-ium chloride (*O*-methyl-fagaronine; C4): This compound was synthesized in an identical manner as that reported for **C1**, at the same scale, with the boronic acid replaced with 510 mg (2.8 mmol) 3,4-dimethoxyphenylboronic acid and compound **11** was replaced with 541 mg (1.75 mmol) of compound **12**. After completion of the three-step reaction sequence, precipitation from ice-cold dilute HCl, and chromatographic purification (95:5 DCM/MeOH), 336 mg (48% over three steps) of **C4** was isolated. MP > 300 °C (lit. 303 °C [11]). The collected NMR data was consistent with that reported from an alternative synthesis of this compound [11].

X-Ray crystallography data

Crystals of variant **B4** were grown via a slow vapor diffusion method, wherein a solution of **B4** in equal parts of dichloromethane and methanol was stored in a pentane vapor chamber for 24 h, resulting in microcrystals of sufficient quality for analysis. Crystals of variant **B6** resulted from a saturated solution of **B6** in methanol, which provided crystals of sufficient quality for analysis.

For **B4**, an arbitrary sphere of data was collected on a yellow blade-like crystal, having approximate dimensions of 0.263 × 0.082 × 0.028 mm. For **B6**, an arbitrary sphere of data was collected on a yellow rod-like crystal, having approximate dimensions of 0.253 × 0.060 ×

0.054 mm. Data were recorded on a Bruker D8 diffractometer equipped with a Bruker PHOTON-III detector using a combination of ω- and φ-scans of 0.5° at an ambient temperature of 120 K [12].

The crystal measured for **B4** was found to be a 180° rotation twin about the reciprocal [0 1 1] direction. The twin domains refined to a 0.495:0.505 ratio.

Data were corrected for absorption and polarization effects and analyzed for space group determination [13]. The structures were solved by iterative methods and expanded routinely [14]. The model was refined by full-matrix least-squares analysis of F2 against all reflections [15]. All non-hydrogen atoms were refined with anisotropic atomic displacement parameters. Unless otherwise noted, hydrogen atoms were included in calculated positions. Atomic displacement parameters for the hydrogens were tied to the equivalent isotropic displacement parameter of the atom to which they are bonded ($U_{iso}(H) = 1.5U_{eq}(C)$ for methyl, $1.2U_{eq}(C)$ for all others).

The crystallographic data are summarized in Table S1. The structures were deposited to Cambridge Crystallographic Data Center with reference CCDC numbers 2271457-2271458

Table S1. Crystal data and structure refinement for **B4** and **B6**.

Identification code	**B4**	**B6**
Empirical formula	$C_{19}H_{17}ClFNO_3$	$C_{43}H_{50}N_2O_{11}$
Formula weight	361.79	770.85
Temperature (K)	120(2)	120(2)
Wavelength (Å)	1.54178	1.54178
Crystal system	Triclinic	Triclinic
Space group	P-1	P-1
(*a*, Å)	5.60980(10)	7.8890(2)
(*b*, Å)	9.8461(2)	12.3476(3)
(*c*, Å)	15.7454(3)	*c* = 19.6989(5)
(α, °)	98.5670(10)	α = 93.0628(9)
(β, °)	99.3100(10)	β = 93.2269(10)
(γ, °)	105.1880(10)	γ = 103.6883(10)
Volume (Å^3)	811.37(3)	1857.00(8)
Z	2	2
Density (calculated, g.cm^{-3})	1.481	1.379
Absorption coefficient (μ, mm^{-1})	2.352	0.817
F(000)	376	820
Crystal color, habit	yellow, blade	yellow, rod
Crystal size (mm^3)	0.263 × 0.082 × 0.028	0.253 × 0.060 × 0.054
θ range for data collection (°)	2.903 to 69.829	2.252 to 69.556
Index ranges	$-6 \leq h \leq 6$, $-11 \leq k \leq 11$, 0	$-9 \leq h \leq 9$, $-14 \leq k \leq 14$, -23

	≤ l ≤ 19	≤ l ≤ 23
Reflections collected	5942	36516
Independent reflections	5942 [R_{int} = 0.000]	6808 [R_{int} = 0.0330]
Completeness to θ = 67.679°	98.3 %	98.4 %
Absorption correction	Semi-empirical from equivalents	Numerical
Max. and min. transmission	0.864348 and 0.756386	1.0000 and 0.8770
Refinement method	Full-matrix least-squares on F^2	Full-matrix least-squares on F^2
Data / restraints / parameters	5942 / 0 / 233	6808 / 0 / 517
Goodness-of-fit on F^2	1.048	1.052
Final R indices [I>2σ(I)]	R_1 = 0.0406, wR_2 = 0.0955	R_1 = 0.0351, wR_2 = 0.0945
R indices (all data)	R_1 = 0.0487, wR_2 = 0.0996	R_1 = 0.0420, wR_2 = 0.1003
Extinction coefficient	n/a	n/a
Largest diff. peak and hole ($e^{-}.Å^{-3}$)	0.189 and -0.229	0.269 and -0.208

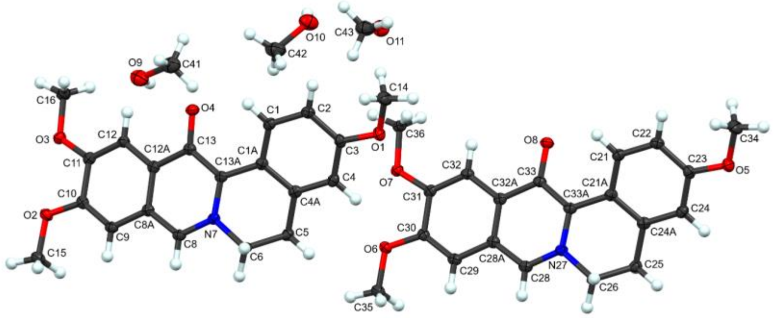

Figure S2: Asymmetric unit of **B6**. Atomic displacement ellipsoids depicted at 50 % probability and hydrogen atoms are spheres of an arbitrary radius.

Proposed mechanism for oxidative by-products

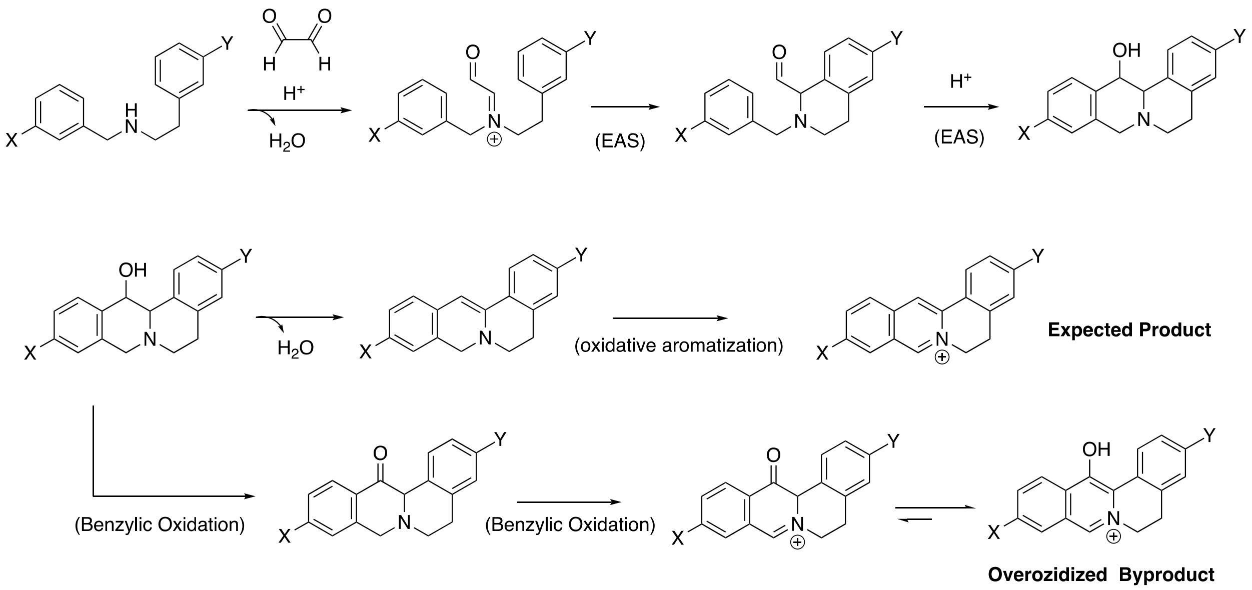

Figure S3: Proposed mechanism explaining the formation of the over-oxidized byproducts **B2**, **B4**, and **B6**. It should be noted the two benzylic-type oxidations may occur in the opposite order.

Antimicrobial disc diffusion assay

Blank antibiotic sensitivity discs (Fisher Scientific, Pittsburgh, PA, USA) were impregnated with 20 μL of a 6 mg/mL solution of the compound of interest (dissolved in methanol, dichloromethane, or a 1:1 mixture of the solvents), resulting in 0.12 of compound per disc. Once the solvent fully evaporated, discs were placed onto a media plate with a lawn of one of the following microorganisms: *Staphylococcus aureus*, *Bacillus cereus, Bacillus subtilis, Staphylococcus epidermidis, Corynebacterium pseudodiphtheriticum*, *Escherichia coli*, *Proteus mirabilis*, *Enterobacter aerogenes*, *Enterobacter cloacae*, *Saccharomyces cerevisiae, Candida albicans* or *Penicillium chrysogenum* (Carolina Biological Supply Company, Burlington, NC, USA). Plates were either incubated at 37 °C (for bacteria) or at room temperature (for fungi). After 48 h of growth, zones of inhibition were measured using a ruler for each disc, and mean zones of inhibition in millimeters were calculated. Vancomycin, streptomycin and/or fluconazole were used as positive controls. The solvents alone (methanol or dichloromethane) were used as negative controls and showed no zones of inhibition. A representative result for the berberine series with *S. aureus* is shown in Figure S4.

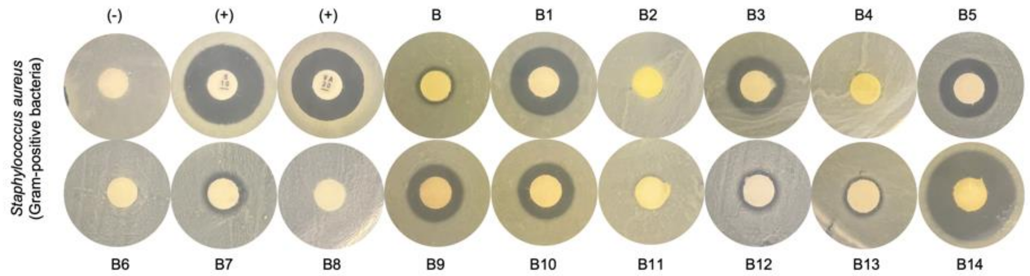

Figure S4: A representative trial showing antimicrobial effects of berberine (**B**) and the berberine variants (**B1**–**B14**) against Gram-positive *S. aureus* bacteria; 20 μL phytocompound treatments at 6 mg/mL were used, resulting in 0.12 mg of compound per disc. Streptomycin and vancomycin were used as positive controls, and solvent was used as the negative control and showed no zone of inhibition.

The statistical significance of differences in zones of inhibition was determined against five Gram-positive bacteria along with two fungal organisms for a selection of berberine variants prepared in methanol compared to zones of inhibition collected for original berberine. This significance in activity between different variant treatments was assessed using a two-tailed T-test analysis, where differences were considered statistically significant with a value of $P \leq 0.05$. This data is summarized in Figure S5 below.

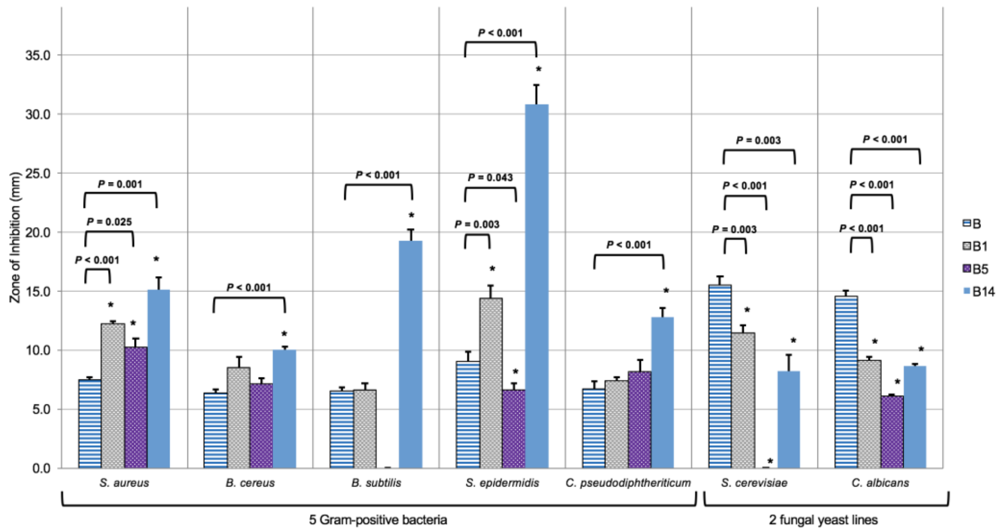

Figure S5: Antimicrobial assay comparison of selected variants to original berberine. Mean zones of inhibition using 0.12 mg of evaporated compound tested against 12 unique microbial species

with the associated standard error. Vancomycin, streptomycin and/or fluconazole were used as positive controls, and solvents were used as negative controls and showed no zones of inhibition.

As some prior publications have reported berberine zones of inhibition using solutions prepared in DMSO, berberine and several variants were re-assessed using DMSO solutions of compounds of interest. Like all antimicrobial assays used in this study, the respective solvent was first allowed to fully evaporate from the disc before testing for biological activity. While most microbes showed no difference in the inhibitory effects for compounds prepared as DMSO solutions, a fairly pronounced improvement in activity was seen against *S. aureus* when using DMSO solutions of the compounds of interest. A representative trial comparing methanol and DMSO solutions of berberine is displayed in Figure S6, and general changes in activities for DMSO solutions of other variants are summarized in Figure S7.

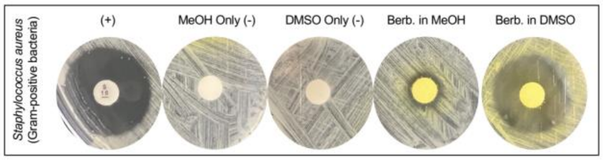

Figure S6: A side-by-side comparison of the difference in activity against Gram-positive *S. aureus* bacteria using methanol solutions of berberine compared to DMSO solutions of berberine; 20 μL phytocompound treatments at 6 mg/mL were used, resulting in 0.12 mg of compound per disc. Streptomycin was used as the positive control, and both solvents were used as the negative controls and showed no zones of inhibition.

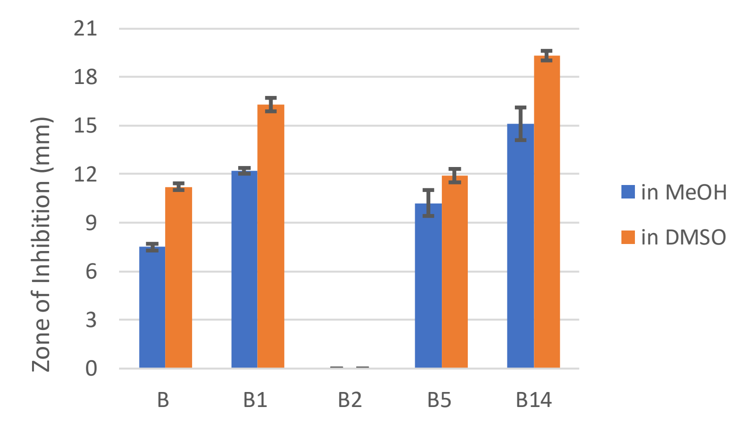

Figure S7: A summary of results showing a consistent improvement in potency against *S. aureus* for selected berberine variants as DMSO solutions when compared to equivalent methanol solutions. Results from **B2** illustrate that inactive variants remained inactive when dissolved in DMSO. Mean zones of inhibition are shown with associated standard error using 20 μL sample at 6 mg/mL in their respective solvent. Streptomycin and vancomycin were used as positive controls, and both solvents were used as the negative controls and showed no zones of inhibition.

<u>Alkaline phosphatase analysis</u>

Selected berberine variants (20 μL at 6 mg/mL in DMSO) were mixed with 1 mL of *S. aureus* cells at a density of 1 × 10^6 CFU/mL. All treatments were placed in a shaking incubator (set to 200 rpm and 35 °C) overnight for ≈ 18 h. The treatments were then centrifuged at 5,000 rpm for 10 min. The resulting supernatants were separated and immediately loaded into a 96-well plate. Color control samples with the berberine variants at the same concentration but no cells were also loaded into the wells as controls. Additionally, supernatants from microbial cells treated with the same volumes of solvent alone were used as negative controls. The treatments were measured for ALP content by the colorimetric Alkaline Phosphatase Assay Kit (ab83369, Abcam, Waltham, Boston, USA) according to the manufacturer's instructions. Results were quantified using a plate reader at 405 nm, and the ALP content normalized for the color control was calculated. The standard deviation was calculated using Microsoft Excel by combining the variation in the color control and experimental treatment for each treatment. Significant differences in the mean ALP content between different treatment groups were assessed using a Mann-Whitney U Test using R-4.2.3 for Windows, where differences were considered statistically significant with a value of $P \leq 0.05$. A summary of results from the berberine variant series is shown in Figure S6 below. A

separate trial, focused only on comparison between berberine (**B**) and variant 14 (**B14**), showed a more pronounced difference in extracellular protein detection, and is displayed in Figure S9. However, the results seen in Figure S9 came from a dataset where the cell density (CFU/mL) of *S. aureus* was unknown, and therefore these results were not incorporated into the summary showed in Figure S8.

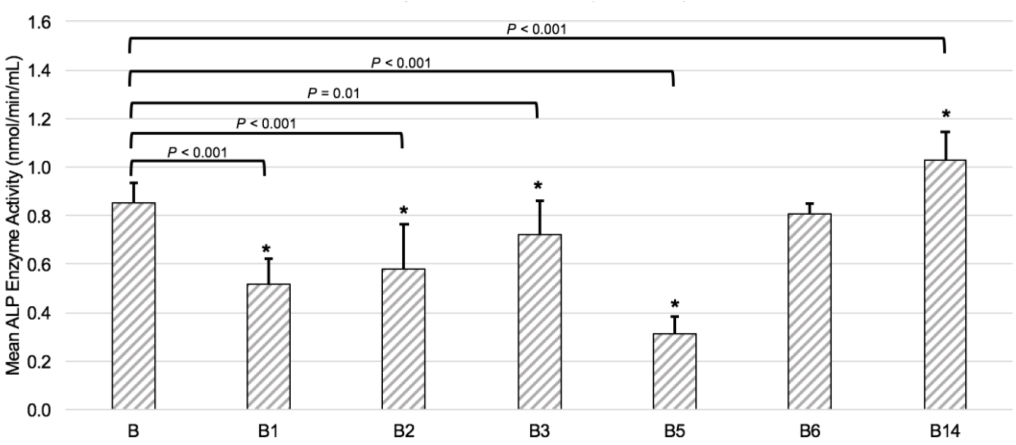

Figure S8: Summary of ALP assay using 20 μL berberine variants (at 6 mg/mL in DMSO) with Gram-positive *S. aureus* bacteria. Mean ALP enzymatic activity is shown with associated standard deviation. Individual treatments were performed overnight (≈ 18 h) using 1 mL of *S. aureus* at a cell density of 1 × 10^6 CFU/mL (1 million cells/mL).

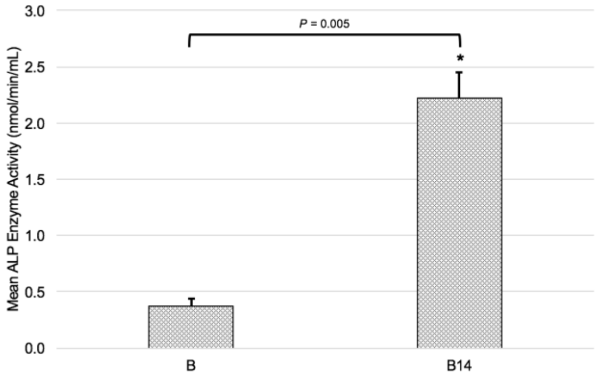

Figure S9: Data from two replicates of an ALP assay comparing original berberine and berberine variant 14 (20 μL at 6 mg/mL in DMSO) with *S. aureus*, $n = 2$ (mean + SD). Note: the cell density (CFU/mL) of *S. aureus* for this experiment was not determined.

Cell culture

Human T84 colon cancer cells were a generous gift from Patrice Bouyer at Valparaiso University, obtained as originally described [16]. All cell lines were maintained and cultured at 37 °C with 5% CO_2, as detailed in [17], using advanced Dulbecco's modified Eagle's medium (ADMEM) (GIBCO-BRL, Grand Island, NY, USA) supplemented with 2% fetal bovine serum (FBS), 1% penicillin/streptomycin mixture, 1% glutamine, and 1% essential amino acids (all obtained from Fisher Scientific, Pittsburgh, PA, USA). Cells were washed with 5 mL of sterile 1X PBS (Fisher Scientific, Pittsburgh, PA, USA) for a 75 cm^2 bottle or with 2 mL for a 25 cm^2 bottle. To detach the monolayer of cells from the surface, 1 mL of trypsin (Fisher Scientific, Pittsburgh, PA, USA) was added and incubated at 37 °C for 5–10 min; to neutralize the trypsin, an equal volume of culture media was added to the trypsin/cell mixture after transfer to a 15 mL falcon tube. Cells were centrifuged at 1,000 rpm for 5 min at room temperature; then the supernatant (media-trypsin) was removed, and 2–4 mL of culture media was added to the pellet, which was carefully mixed to resuspend. Next, a previously calculated volume of cells was added to a 75 cm^2 bottle with 10 mL of culture media or to a 25 cm^2 bottle with 5 mL of culture media. Cells were incubated at 37 °C with 5% CO_2 until they reached 90% confluency.

Variant treatments and viability assay of colon cancer cells

In 24-well plates, cells were seeded at a density of 30,000 cells per well for T84 cells. To compare the effects of different compounds, T84 cells were treated for 1 h using 20 μL of a 6 mg/mL solution (in either MeOH, or 1:1 MeOH/DCM) of the compound of interest for the berberine series, and 5 μL of a 6 mg/mL solution (in MeOH) of the compound of interest for the chelerythrine series. The same volume of the respective solvent alone was used as a negative control. Cells were then subjected to the Vybrant MTT Cell Proliferation Assay Kit (Molecular Probes, Eugene, OR, USA) to assess cell metabolic activity. Results were quantified using a plate reader at 570 nm, and the mean percentage of viable cells normalized to the control (treatment with the corresponding solvent alone) was calculated.

Copies of ^{1}H and ^{13}C spectra of new compounds:

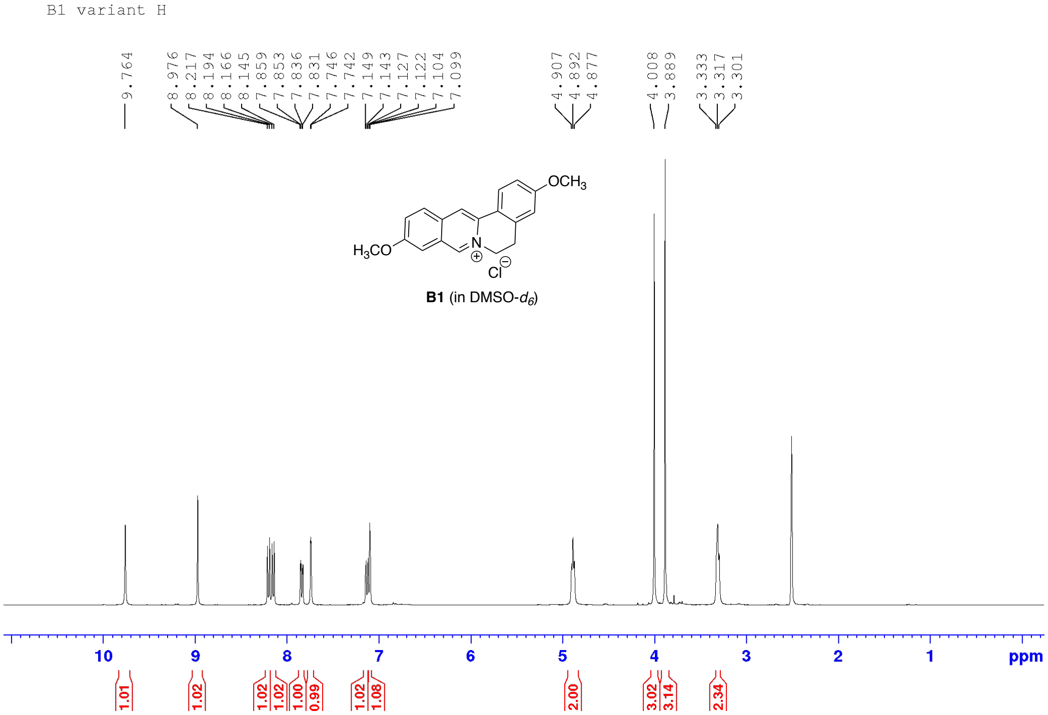

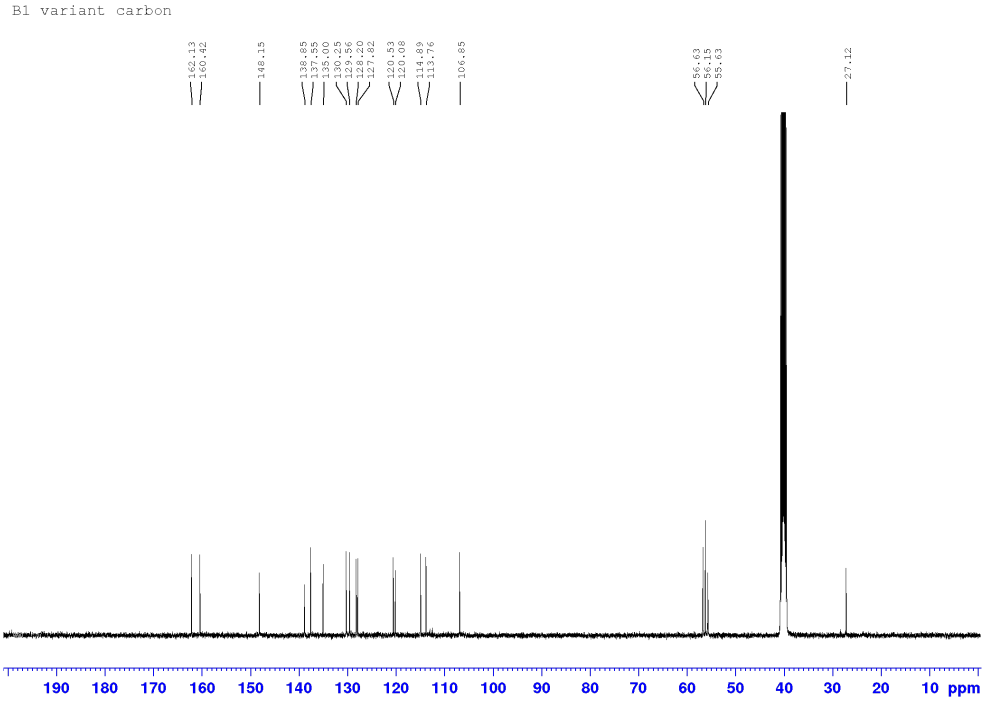

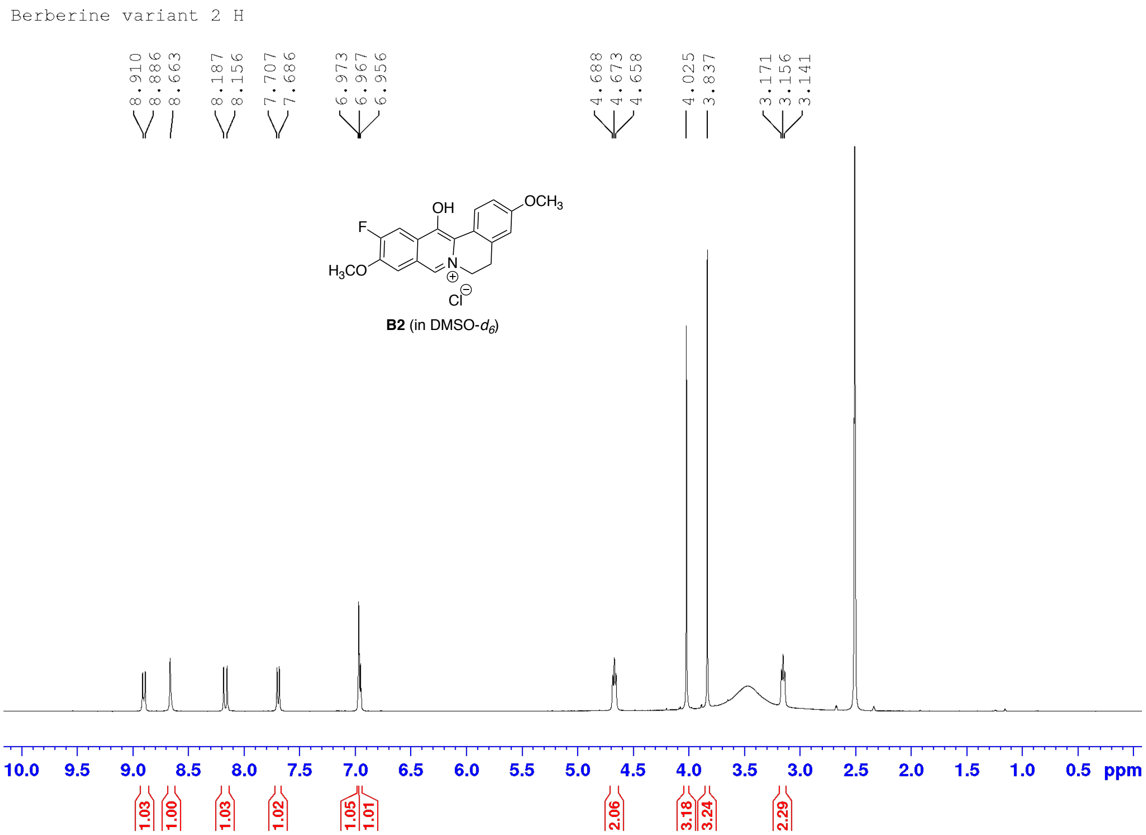
Berberine variant 2 H
8.910
8.886
8.663
8.187
8.156
7.707
7.686
6.973
6.967
6.956
4.688
4.673
4.658
4.025
3.837
3.171
3.156
3.141
OH
OCH3
F
H3CO
N
Cl
B2 (in DMSO-d6)
10.0 9.5 9.0 8.5 8.0 7.5 7.0 6.5 6.0 5.5 5.0 4.5 4.0 3.5 3.0 2.5 2.0 1.5 1.0 0.5 ppm
1.03
1.00
1.03
1.02
1.05
1.01
2.06
3.18
3.24
2.29

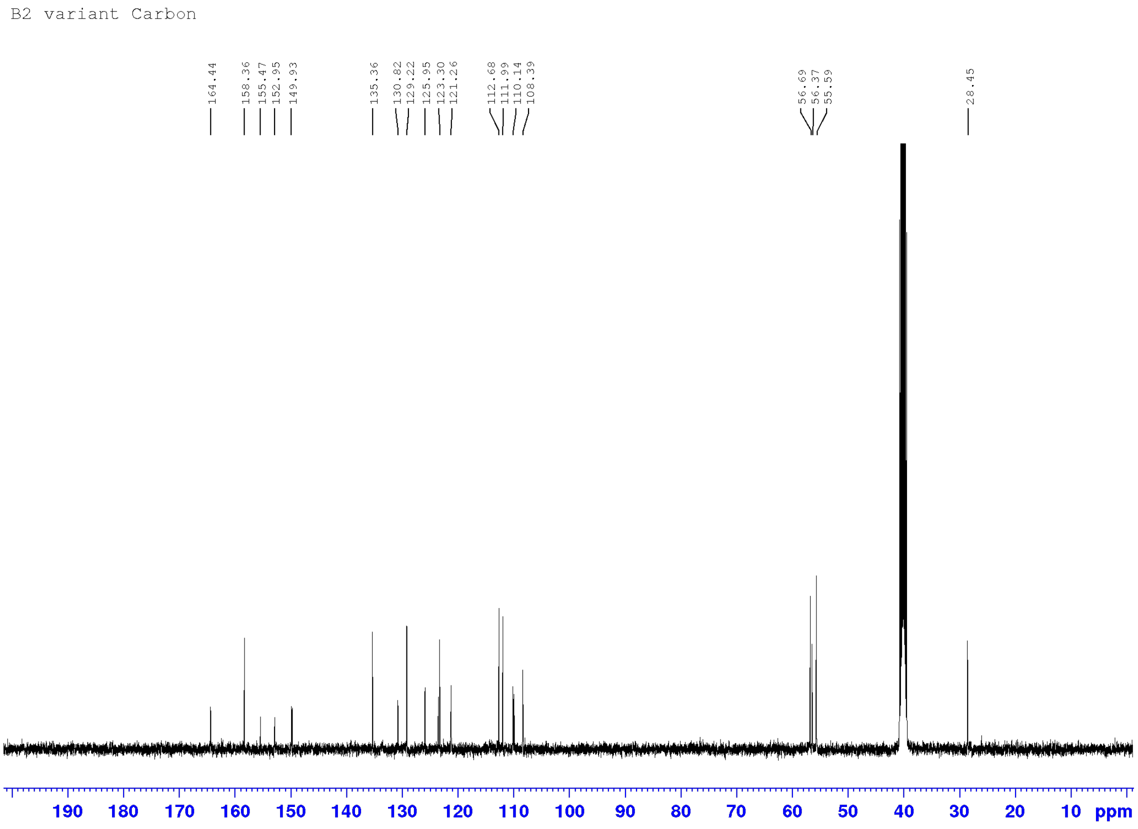
B2 variant Carbon
164.44
158.36
155.47
152.95
149.93
135.36
130.82
129.22
125.95
123.30
121.26
112.68
111.99
110.14
108.39
56.69
56.37
55.59
28.45
190 180 170 160 150 140 130 120 110 100 90 80 70 60 50 40 30 20 10 ppm

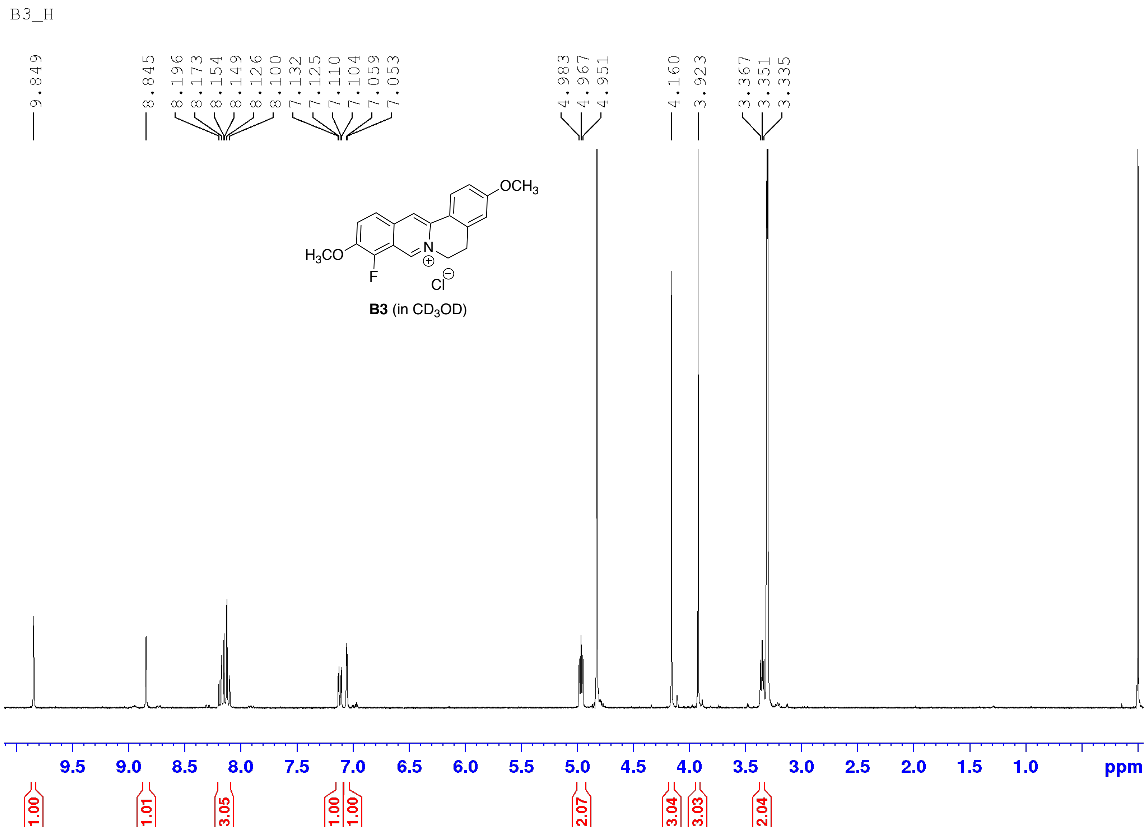
B3_H
9.849
8.845
8.196
8.173
8.154
8.149
8.126
8.100
7.132
7.125
7.110
7.104
7.059
7.053
4.983
4.967
4.951
4.160
3.923
3.367
3.351
3.335
OCH3
H3CO
F
N
Cl
B3 (in CD3OD)
9.5 9.0 8.5 8.0 7.5 7.0 6.5 6.0 5.5 5.0 4.5 4.0 3.5 3.0 2.5 2.0 1.5 1.0 ppm
1.00
1.01
3.05
1.00
1.00
2.07
3.04
3.03
2.04

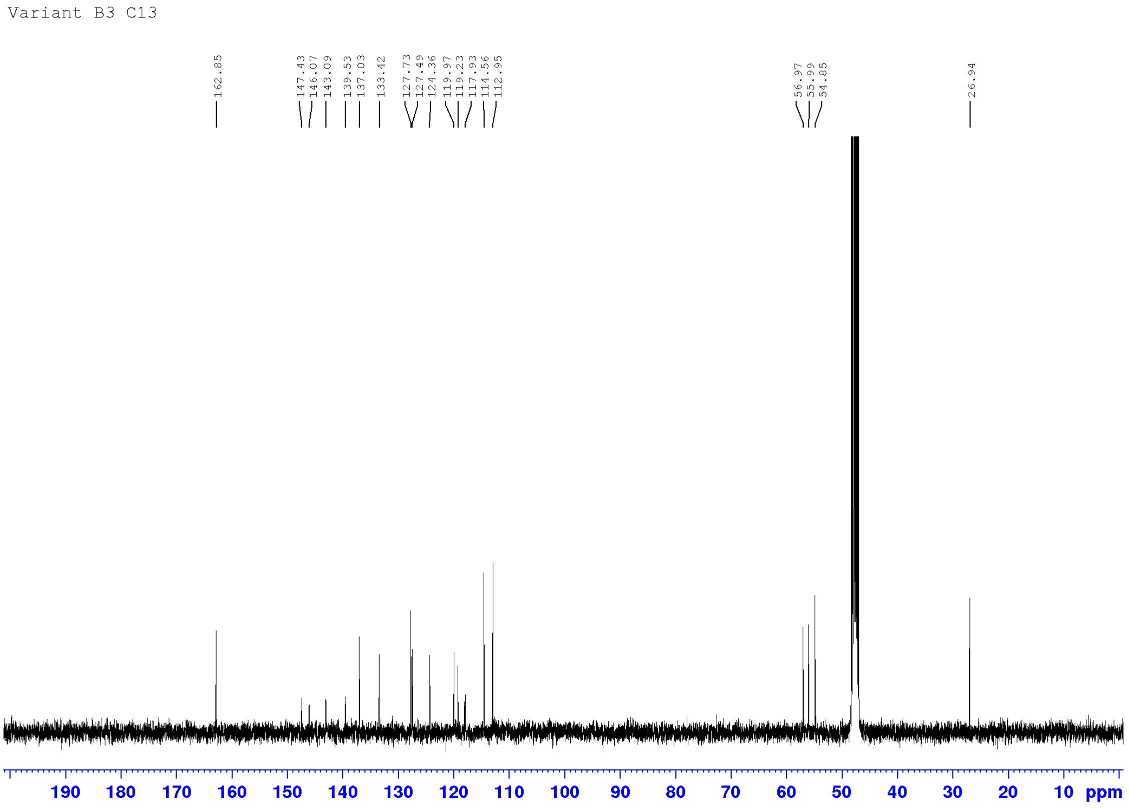
Variant B3 C13
162.85
147.43
146.07
143.09
139.53
137.03
133.42
127.73
127.49
124.36
119.97
119.23
117.93
114.56
112.95
56.97
55.99
54.85
26.94
190 180 170 160 150 140 130 120 110 100 90 80 70 60 50 40 30 20 10 ppm

B4_H

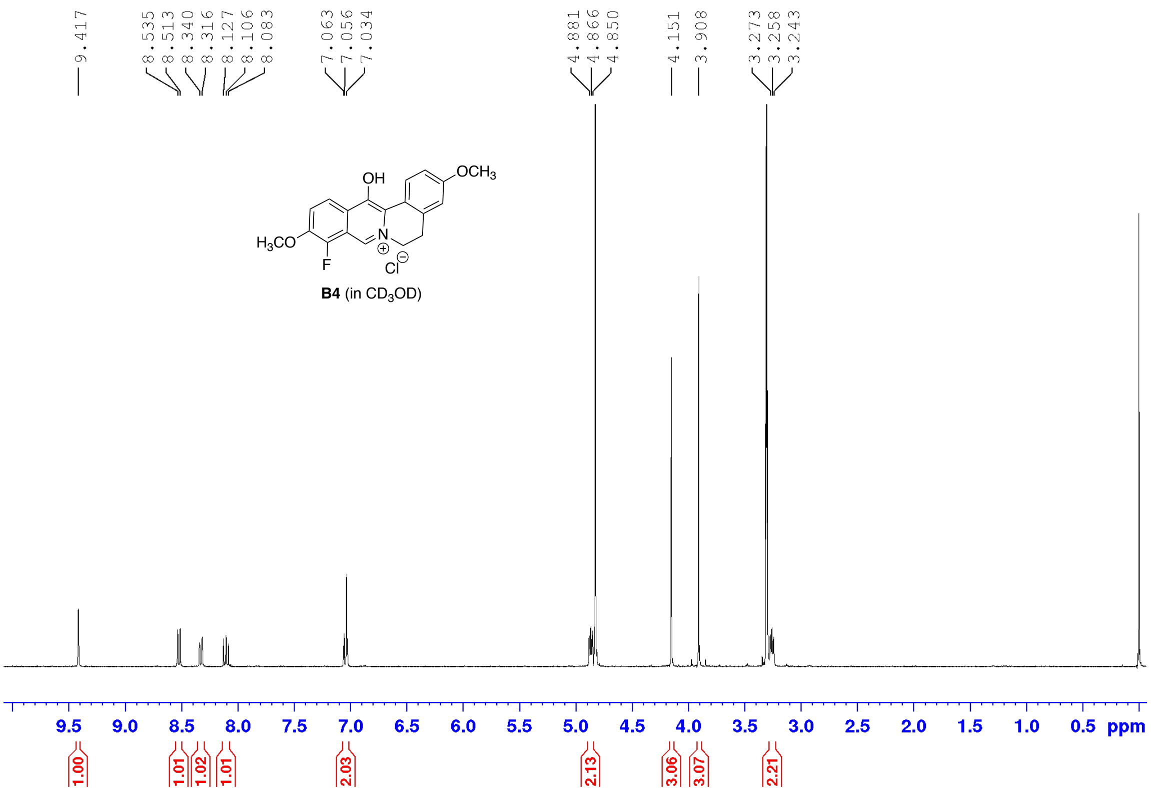

B4 variant C13

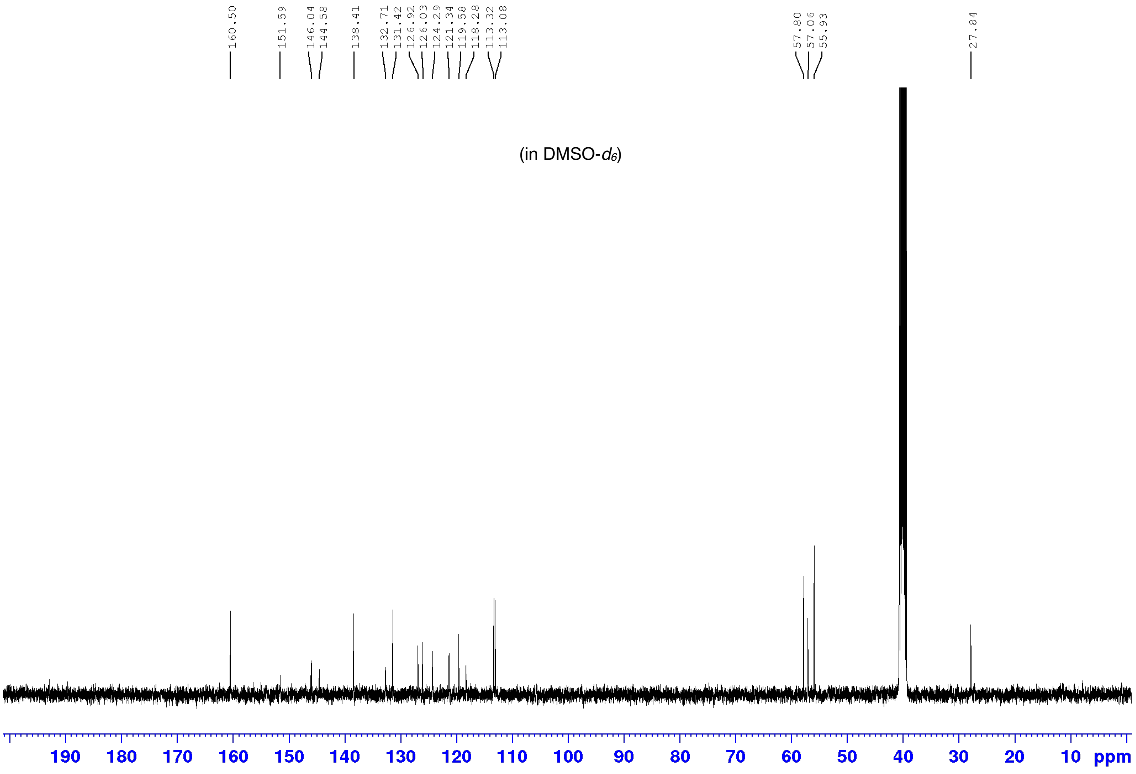

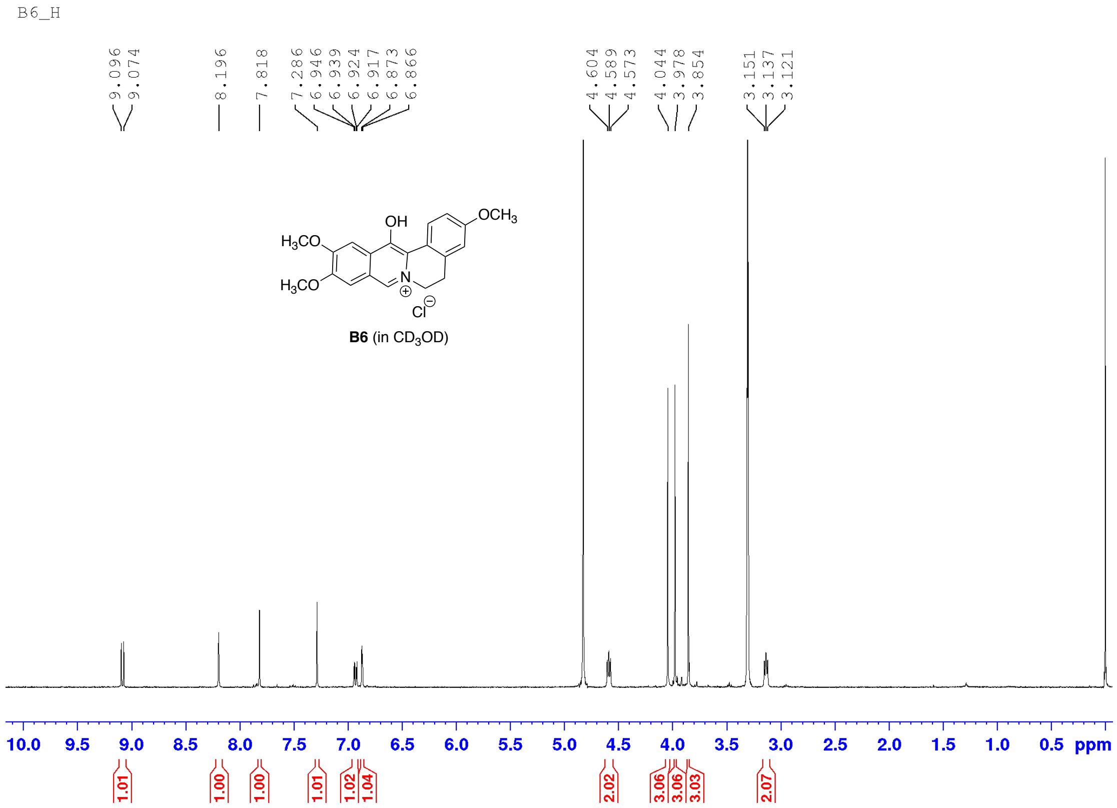
B6_H
9.096
9.074
8.196
7.818
7.286
6.946
6.939
6.924
6.917
6.873
6.866
4.604
4.589
4.573
4.044
3.978
3.854
3.151
3.137
3.121
OH
OCH3
H3CO
H3CO
N
Cl
B6 (in CD3OD)
10.0
9.5
9.0
8.5
8.0
7.5
7.0
6.5
6.0
5.5
5.0
4.5
4.0
3.5
3.0
2.5
2.0
1.5
1.0
0.5
ppm
1.01
1.00
1.00
1.01
1.02
1.04
2.02
3.06
3.06
3.03
2.07

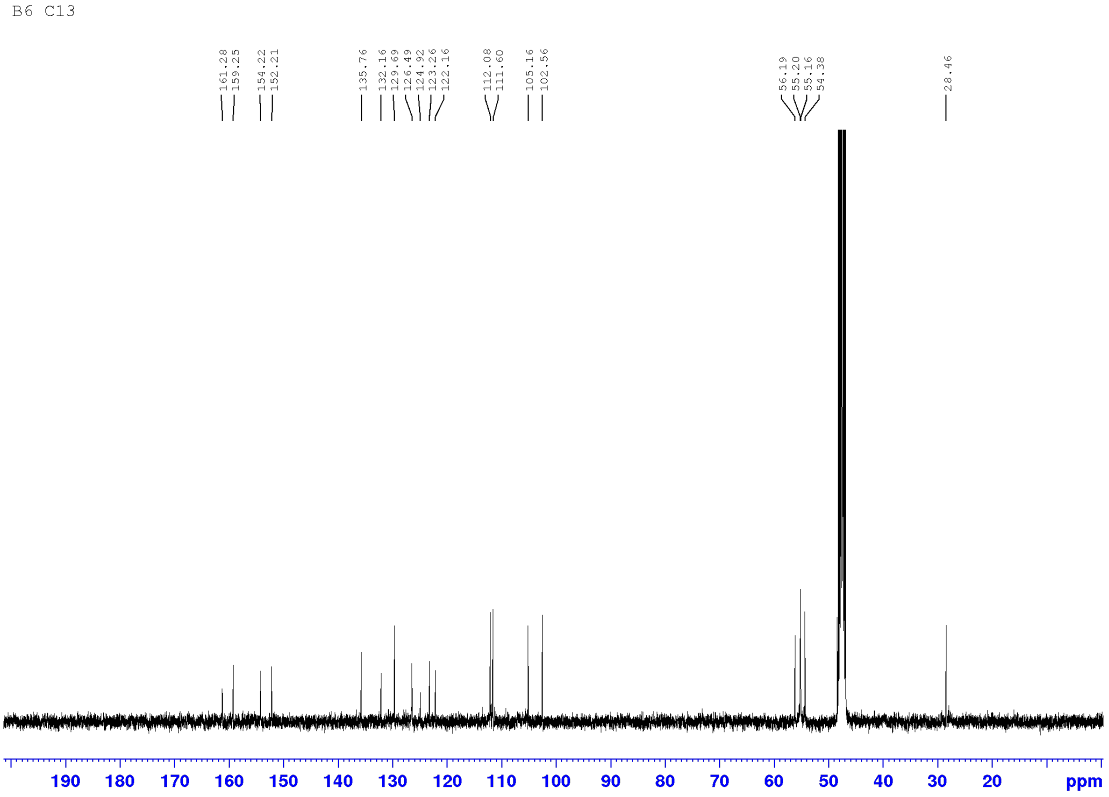
B6 C13
161.28
159.25
154.22
152.21
135.76
132.16
129.69
126.49
124.92
123.26
122.16
112.08
111.60
105.16
102.56
56.19
55.20
55.16
54.38
28.46
190
180
170
160
150
140
130
120
110
100
90
80
70
60
50
40
30
20
ppm

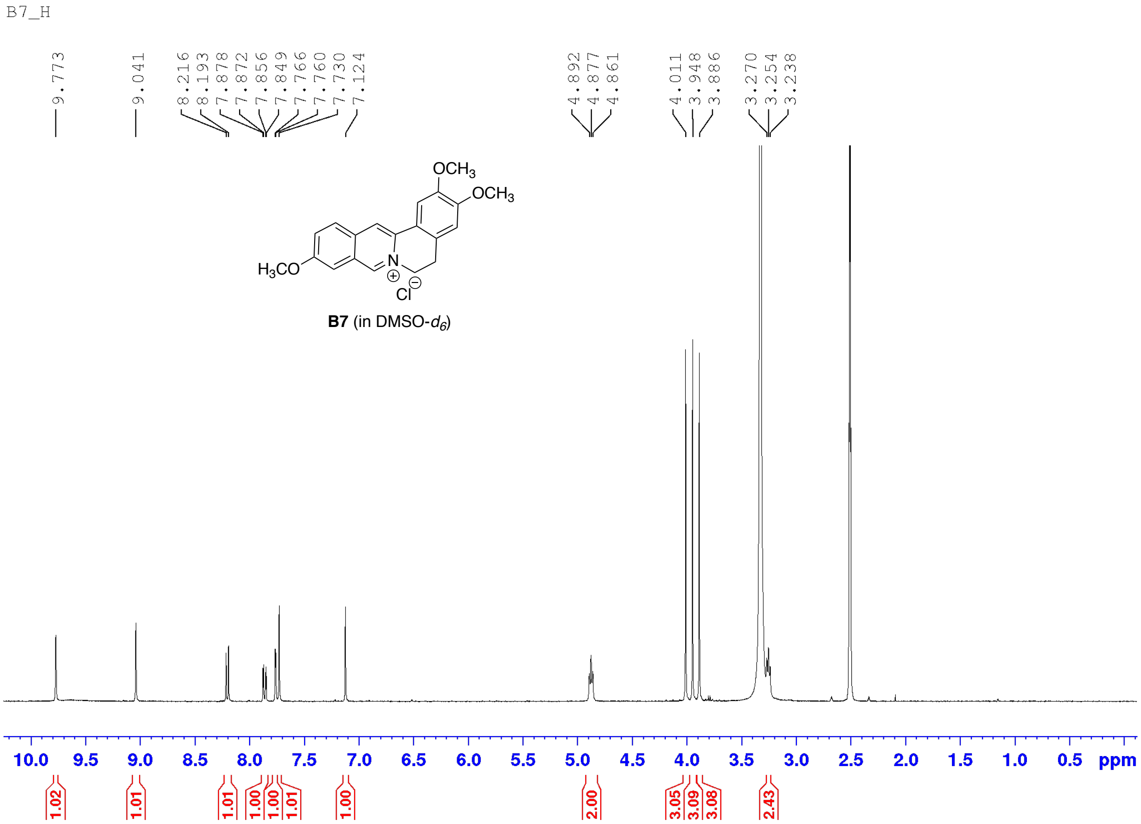
B7_H
9.773
9.041
8.216
8.193
7.878
7.872
7.856
7.849
7.766
7.760
7.730
7.124
4.892
4.877
4.861
4.011
3.948
3.886
3.270
3.254
3.238
OCH3
OCH3
H3CO
N
Cl
B7 (in DMSO-d6)
10.0 9.5 9.0 8.5 8.0 7.5 7.0 6.5 6.0 5.5 5.0 4.5 4.0 3.5 3.0 2.5 2.0 1.5 1.0 0.5 ppm
1.02
1.01
1.01
1.00
1.00
1.01
1.00
2.00
3.05
3.09
3.08
2.43

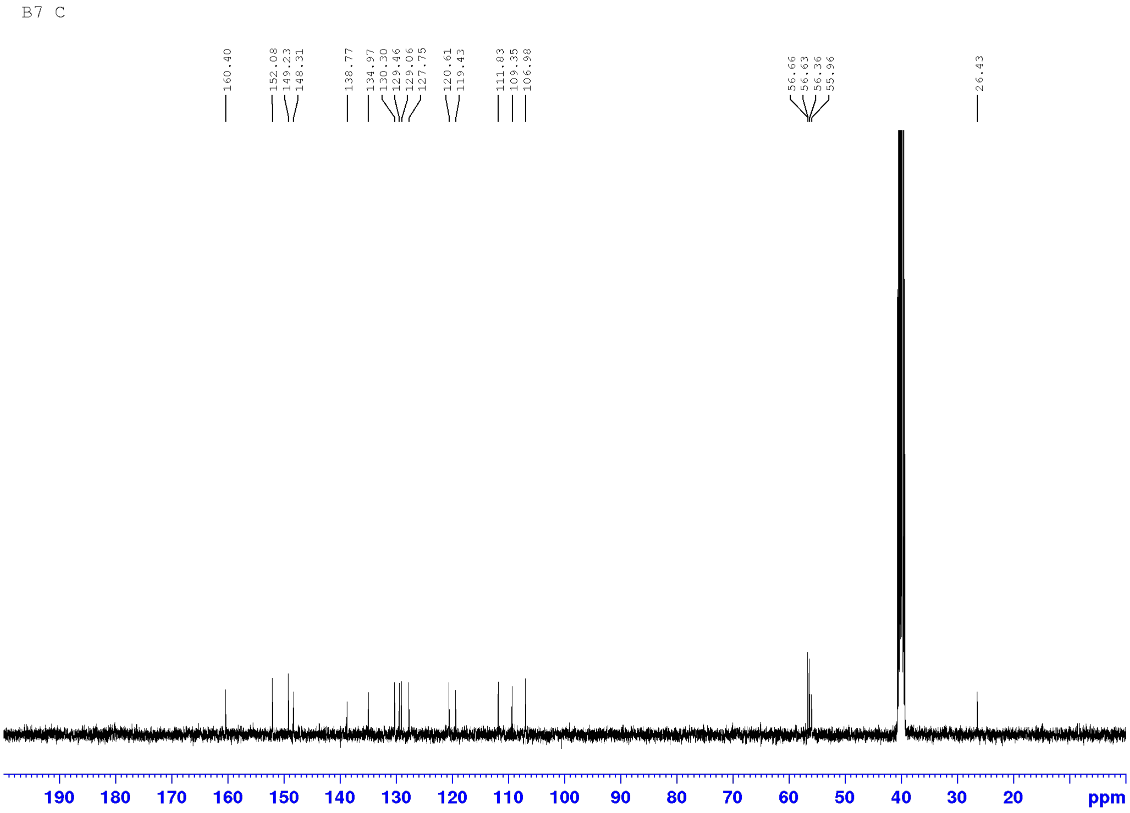
B7 C
160.40
152.08
149.23
148.31
138.77
134.97
130.30
129.46
129.06
127.75
120.61
119.43
111.83
109.35
106.98
56.66
56.63
56.36
55.96
26.43
190 180 170 160 150 140 130 120 110 100 90 80 70 60 50 40 30 20 ppm

B Variant 12

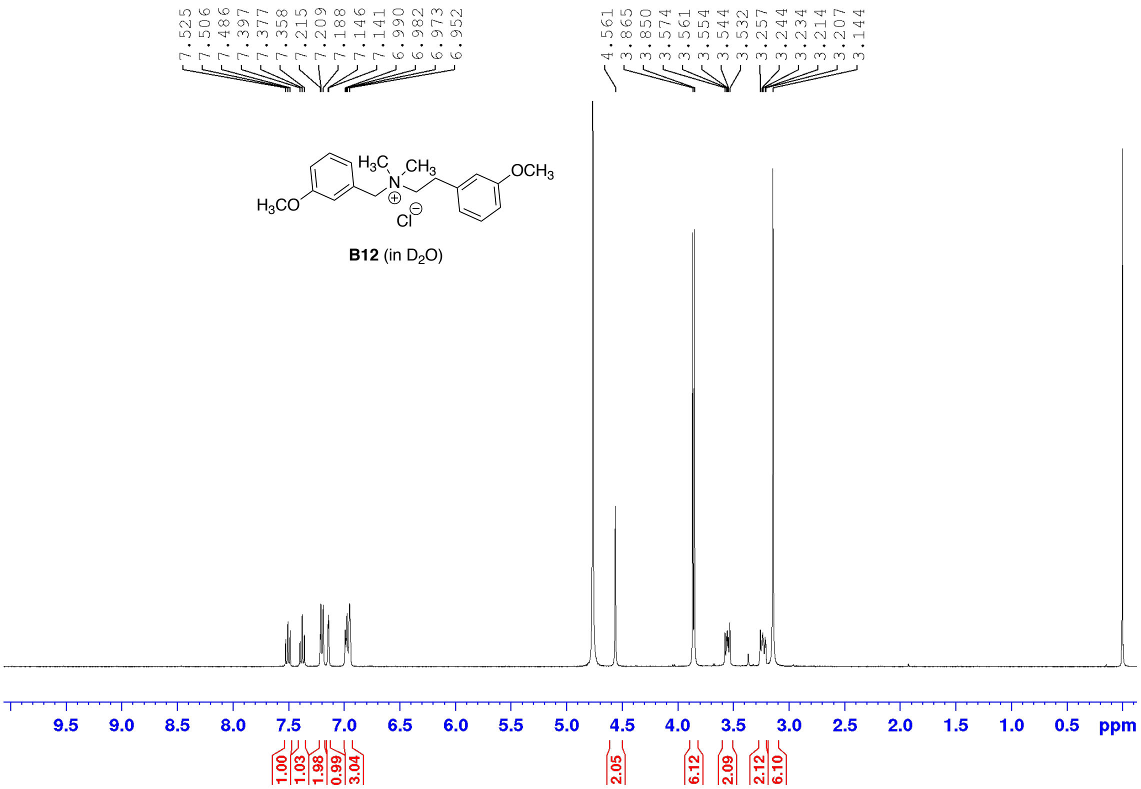

B12 carbon

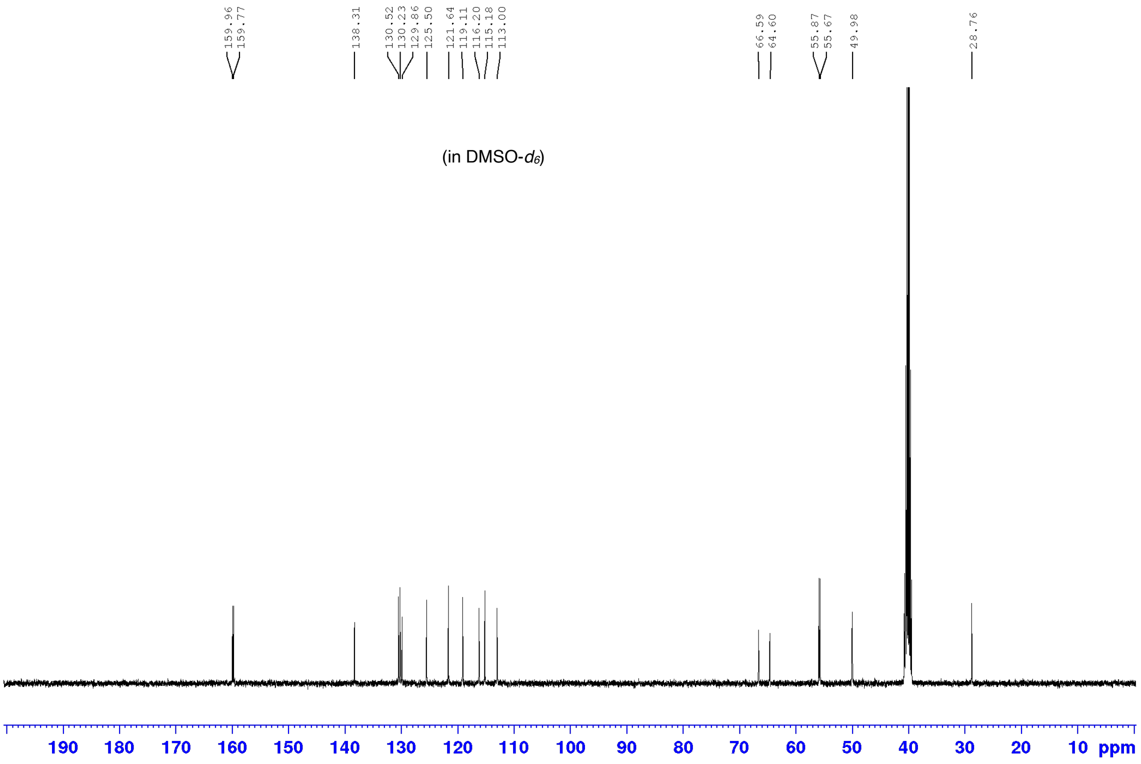

B13 variant proton

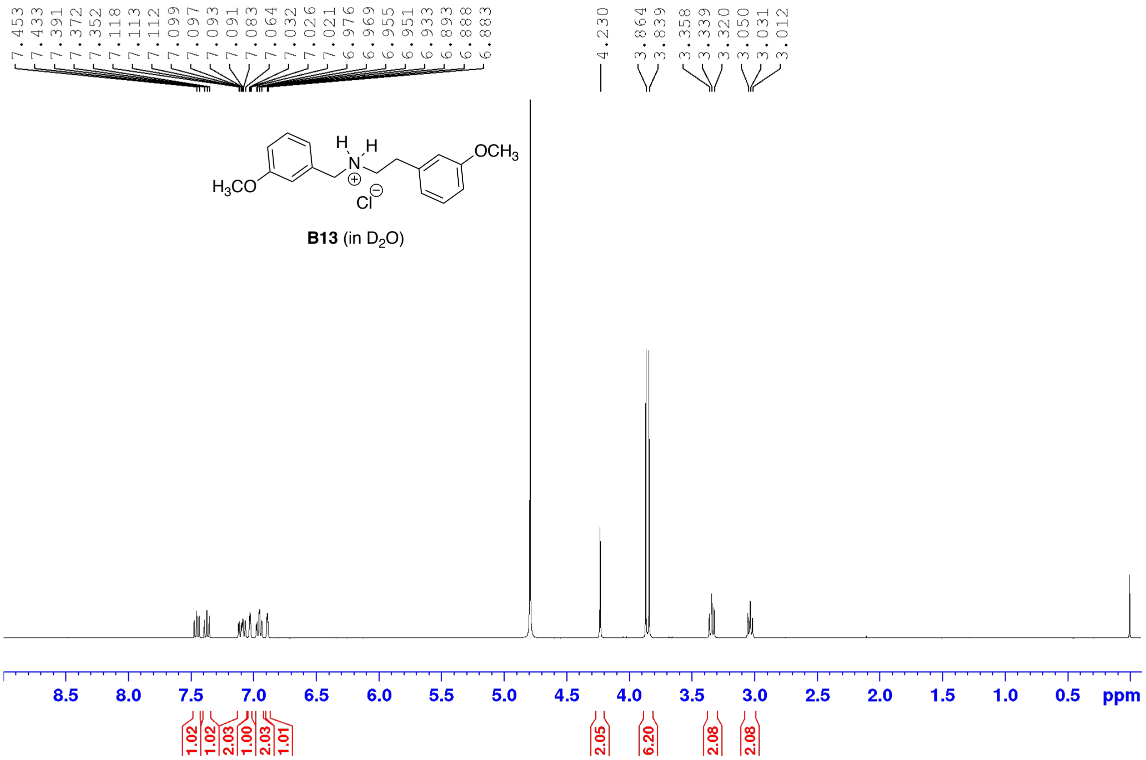

B13 variant Carbon D20

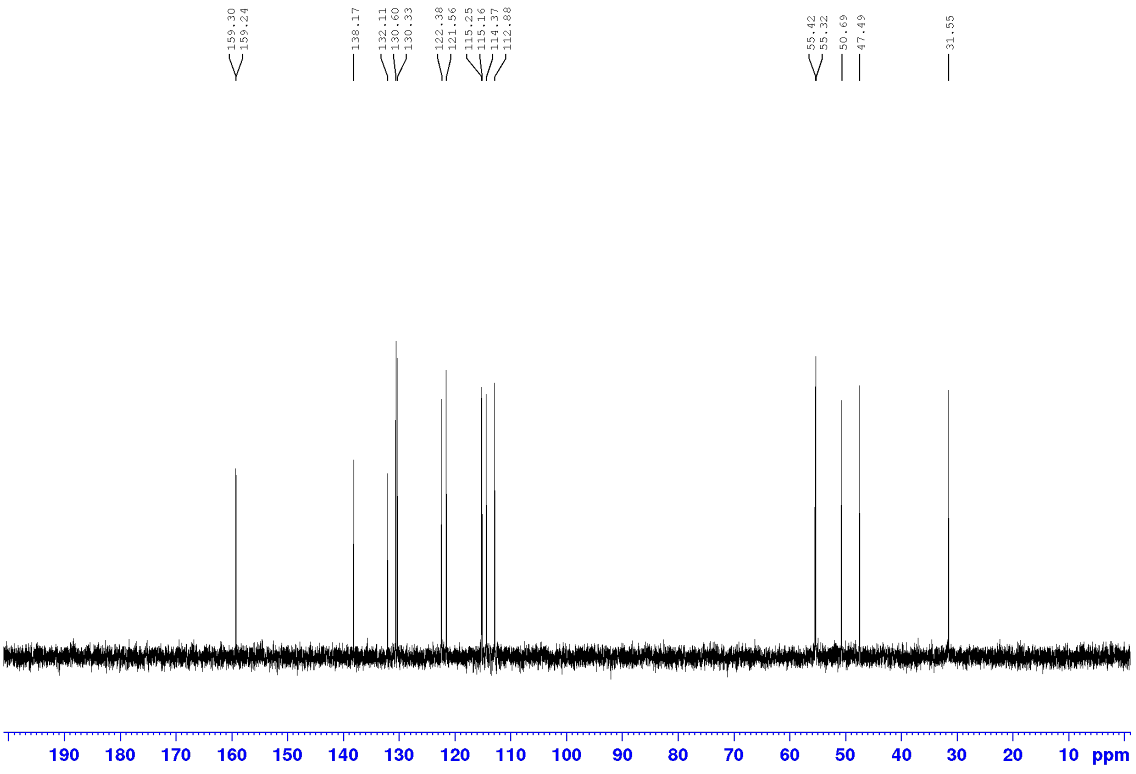

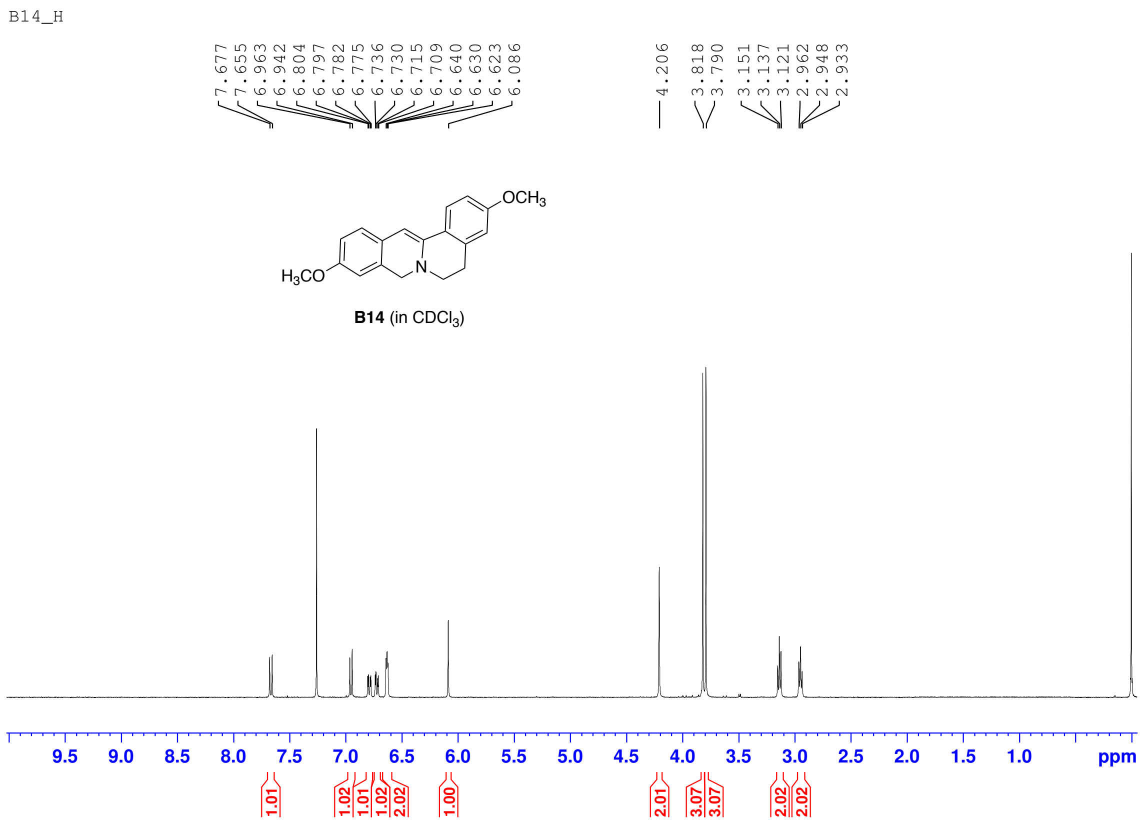
B14_H
7.677 7.655 6.963 6.942 6.804 6.797 6.782 6.775 6.736 6.730 6.715 6.709 6.640 6.630 6.623 6.086
4.206 3.818 3.790 3.151 3.137 3.121 2.962 2.948 2.933
OCH3
H3CO
N
B14 (in CDCl3)
9.5 9.0 8.5 8.0 7.5 7.0 6.5 6.0 5.5 5.0 4.5 4.0 3.5 3.0 2.5 2.0 1.5 1.0 ppm
1.01 1.02 1.01 1.02 2.02 1.00 2.01 3.07 3.07 2.02 2.02

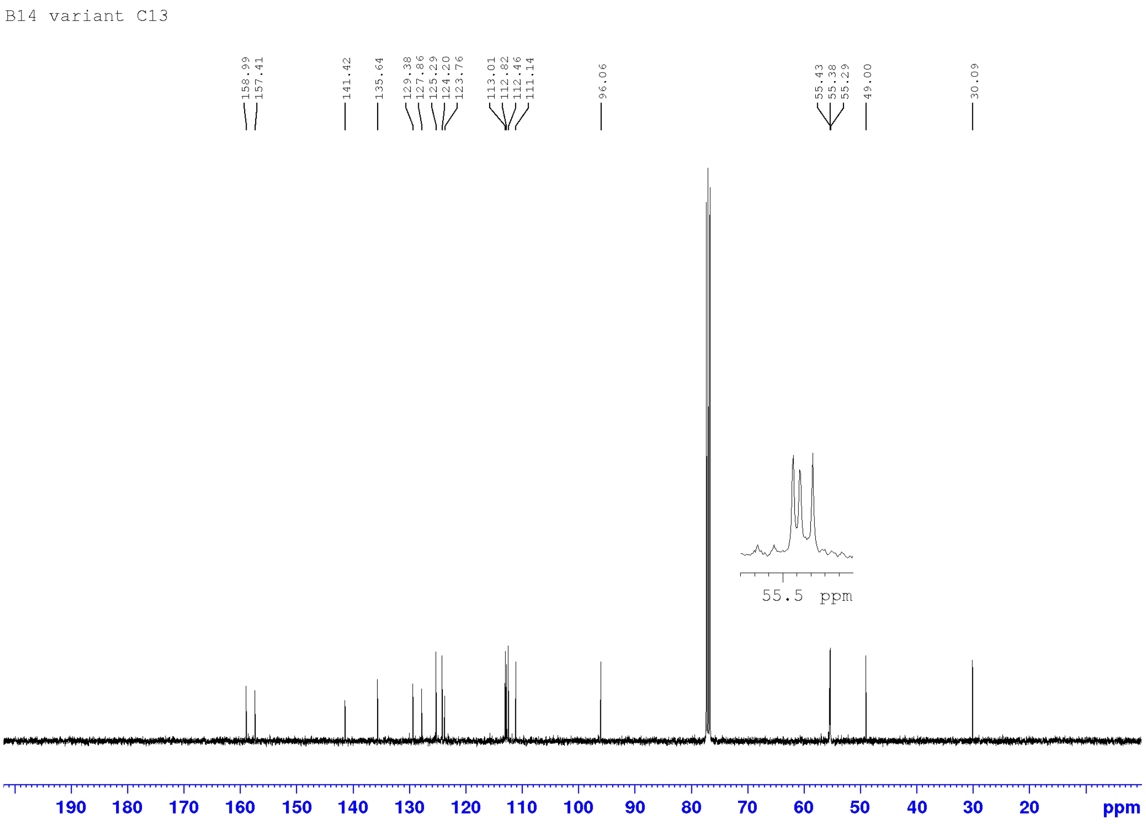
B14 variant C13
158.99 157.41 141.42 135.64 129.38 127.86 125.29 124.20 123.76 113.01 112.82 112.46 111.14 96.06 55.43 55.38 55.29 49.00 30.09
55.5 ppm
190 180 170 160 150 140 130 120 110 100 90 80 70 60 50 40 30 20 ppm

Smiles rearrangement H

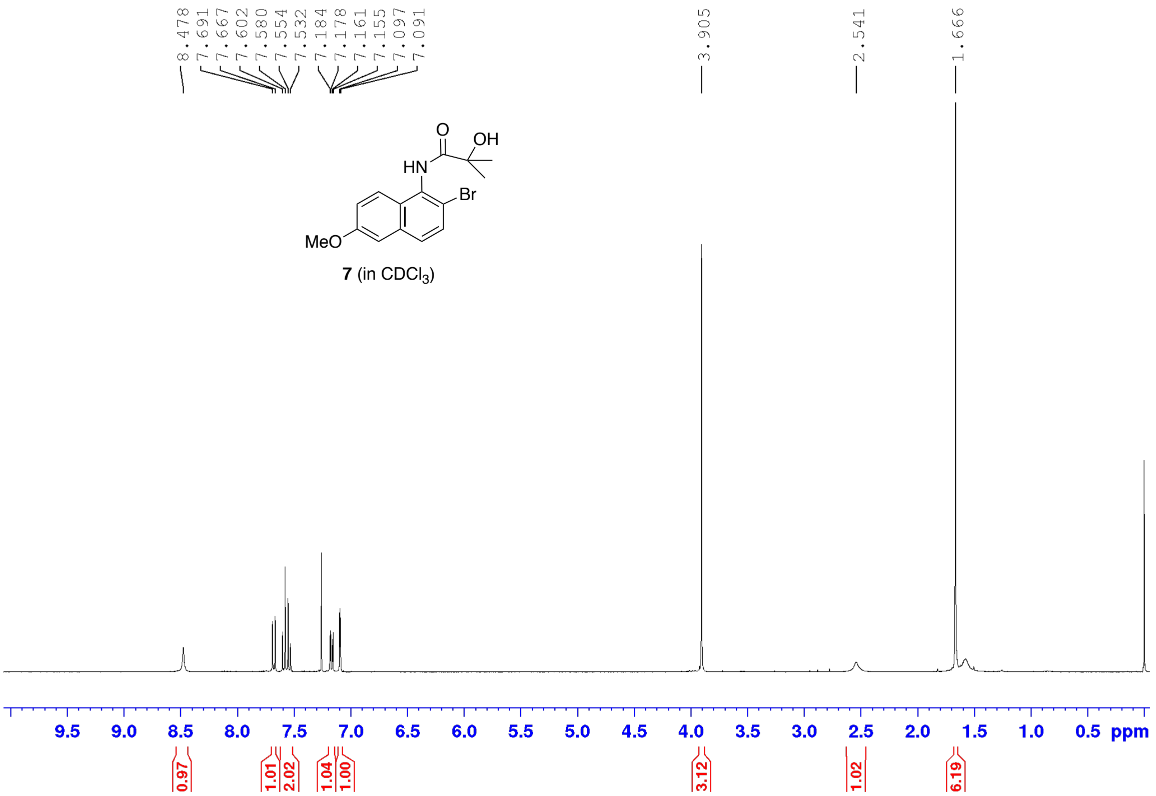

Smiles rearrangement C

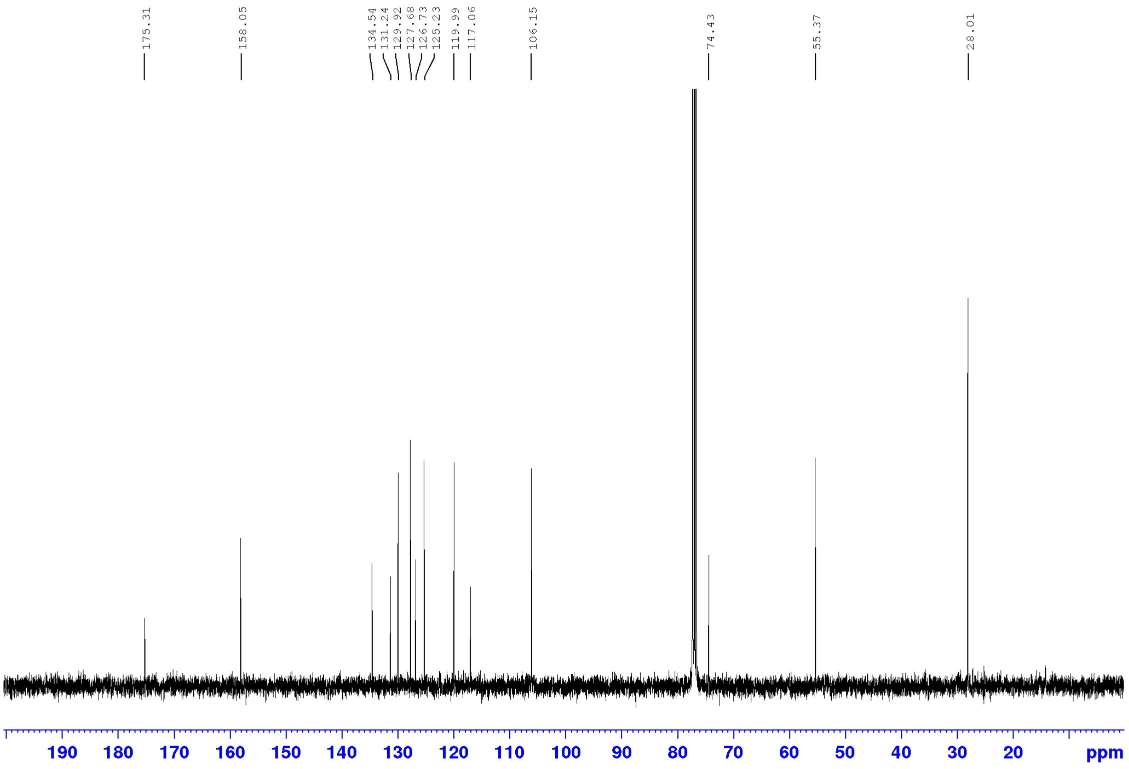

6-methoxy-naphthylamine H

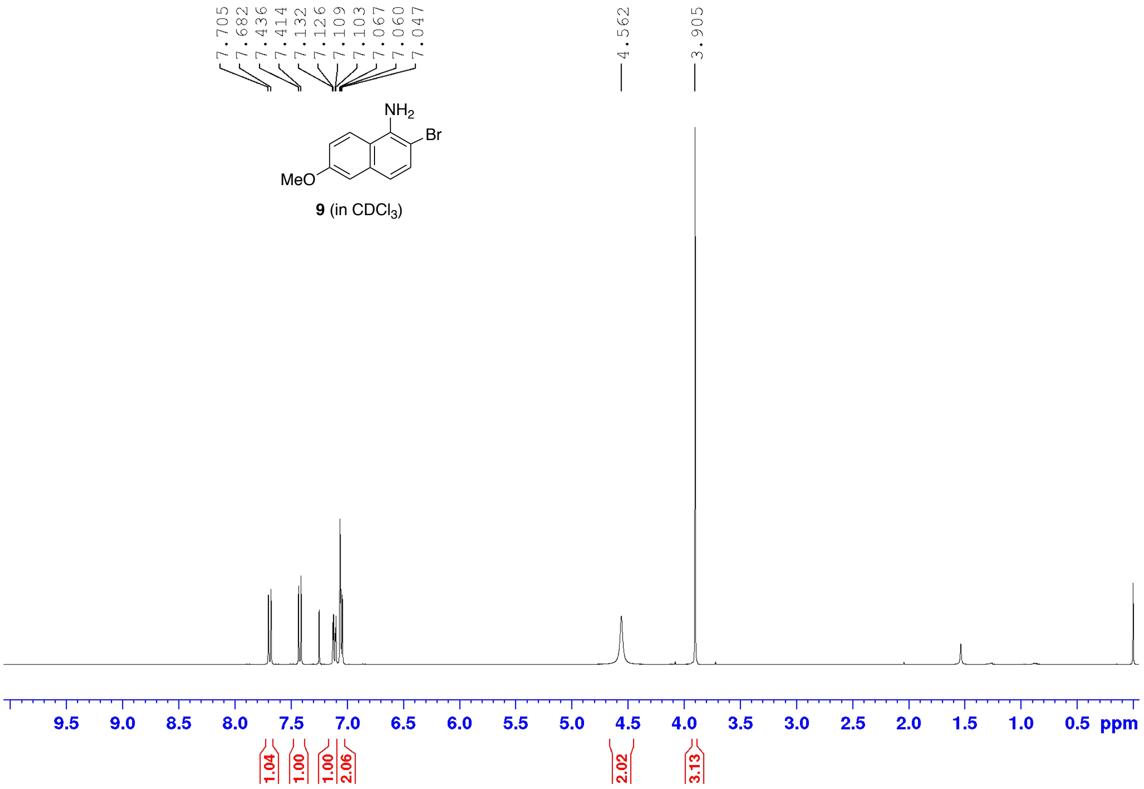

6-methoxy-naphthylamine C

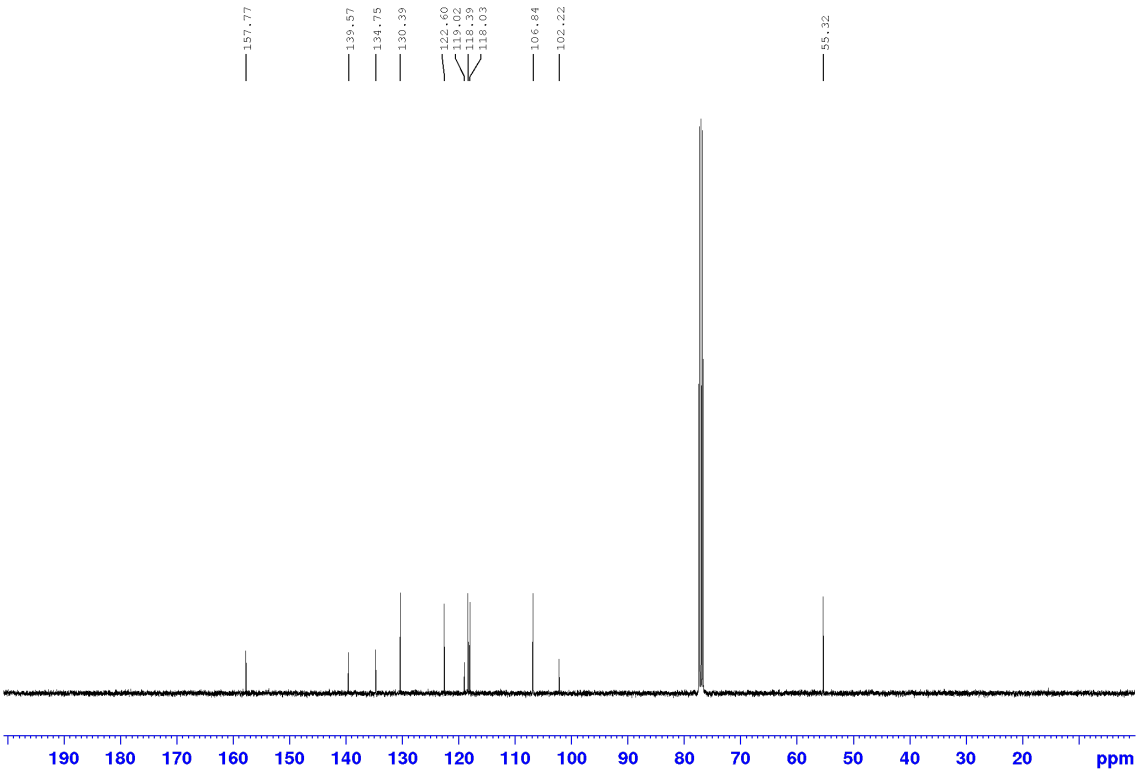

N-formyl-2-bromo-6-methoxy-naphthylamine

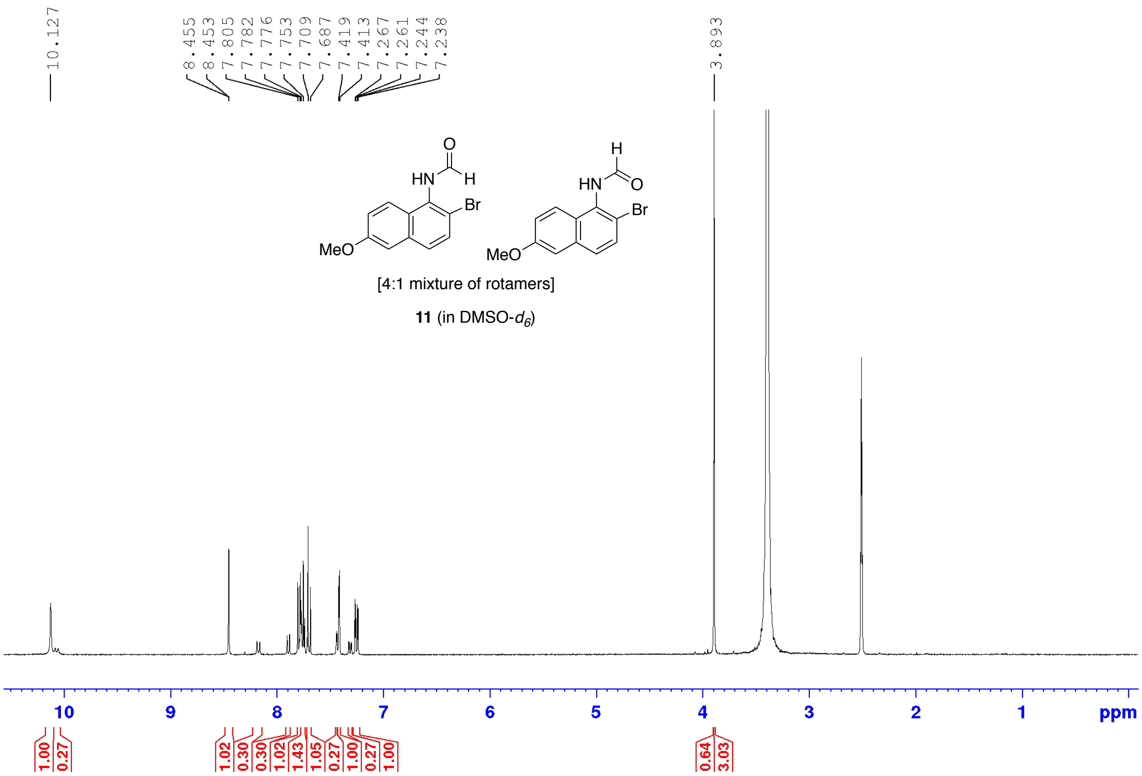

N-formyl-2-bromo-6-methoxy-naphthylamine C13

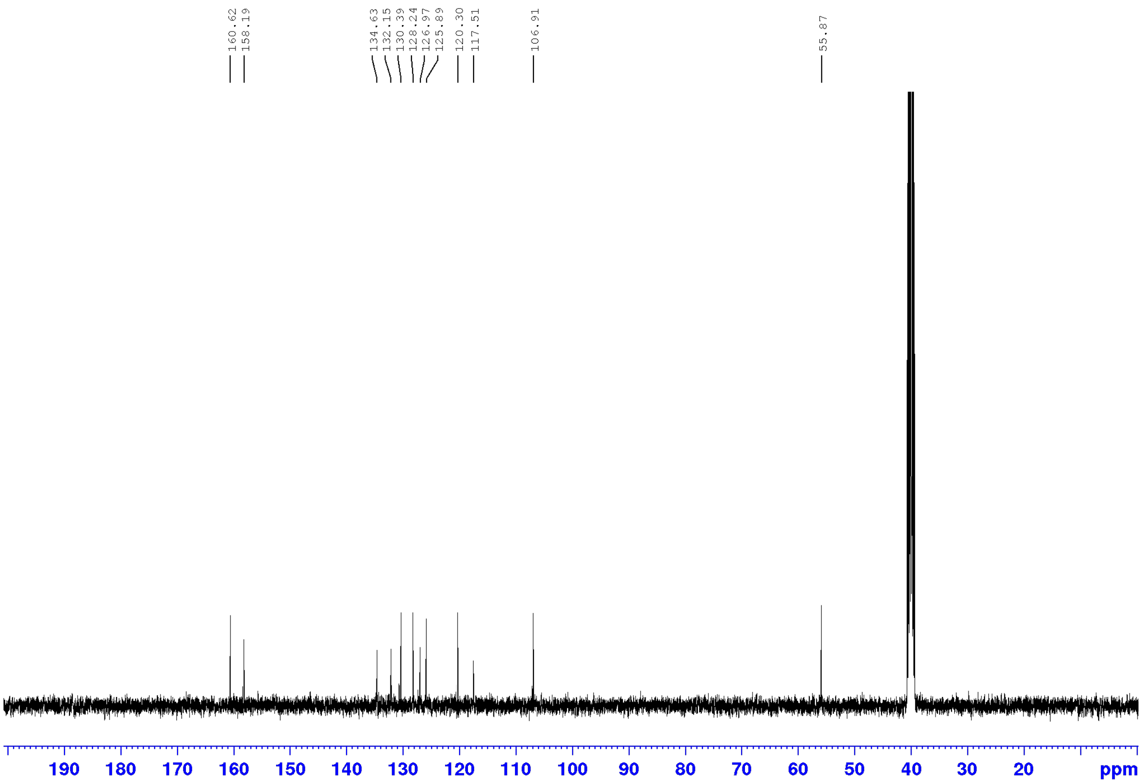

C1_H

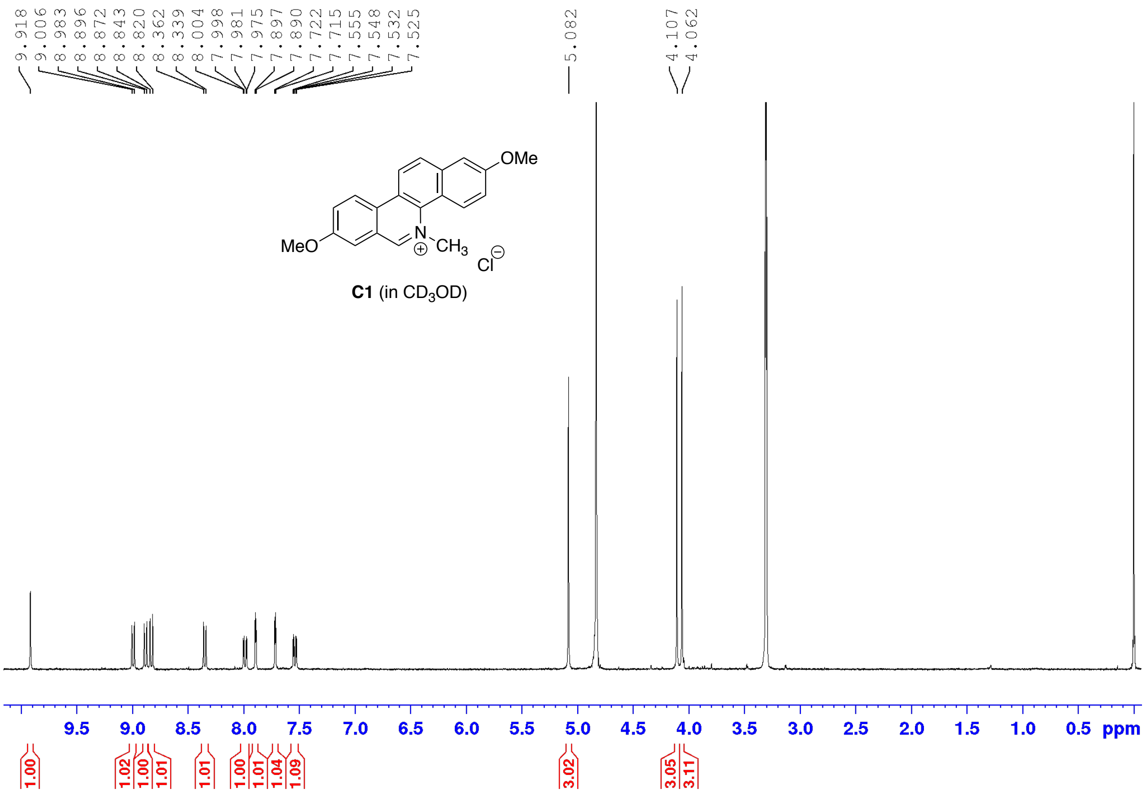

Chelerythrine Variant 1 C13

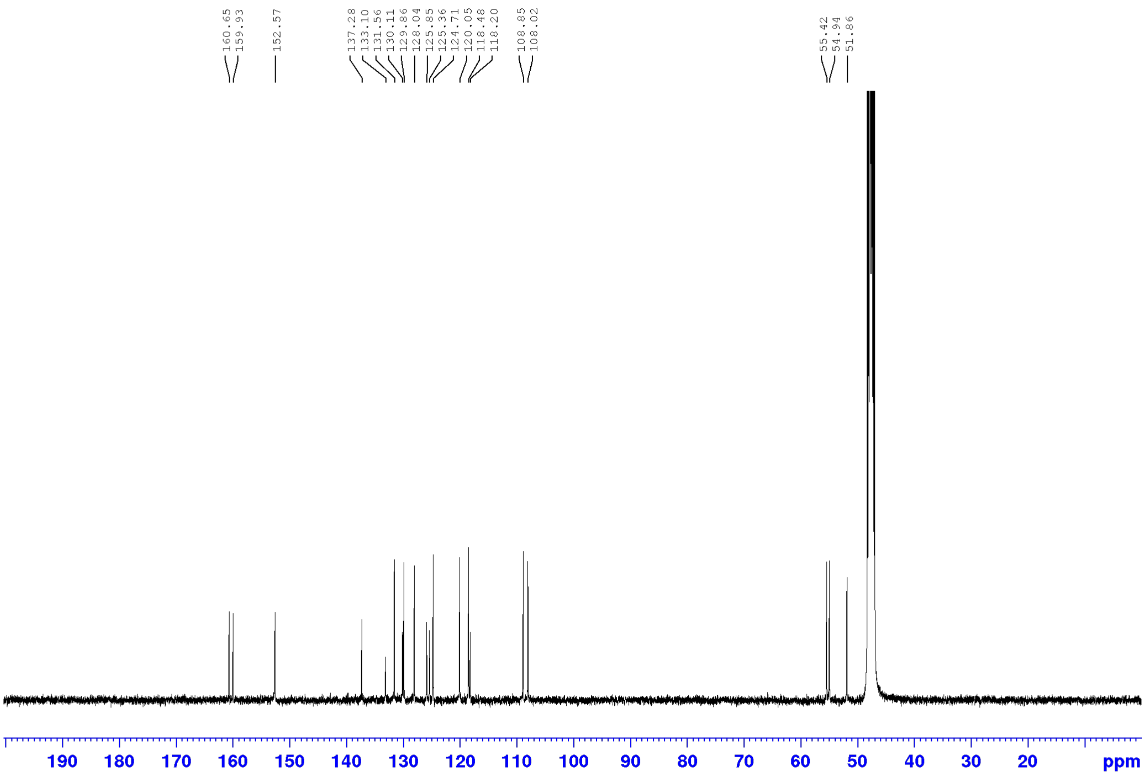

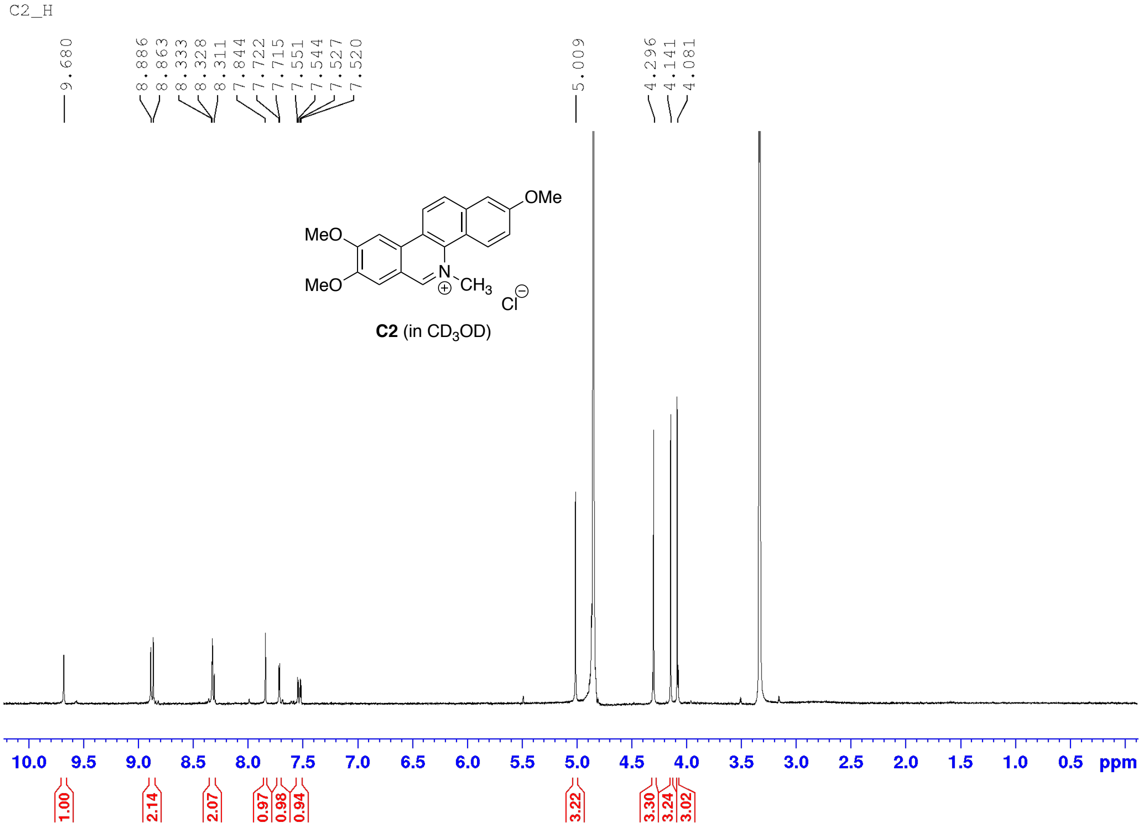
C2_H
9.680
8.886
8.863
8.333
8.328
8.311
7.844
7.722
7.715
7.551
7.544
7.527
7.520
5.009
4.296
4.141
4.081
MeO
MeO
OMe
N
CH3
Cl
C2 (in CD3OD)
10.0 9.5 9.0 8.5 8.0 7.5 7.0 6.5 6.0 5.5 5.0 4.5 4.0 3.5 3.0 2.5 2.0 1.5 1.0 0.5 ppm
1.00
2.14
2.07
0.97
0.98
0.94
3.22
3.30
3.24
3.02

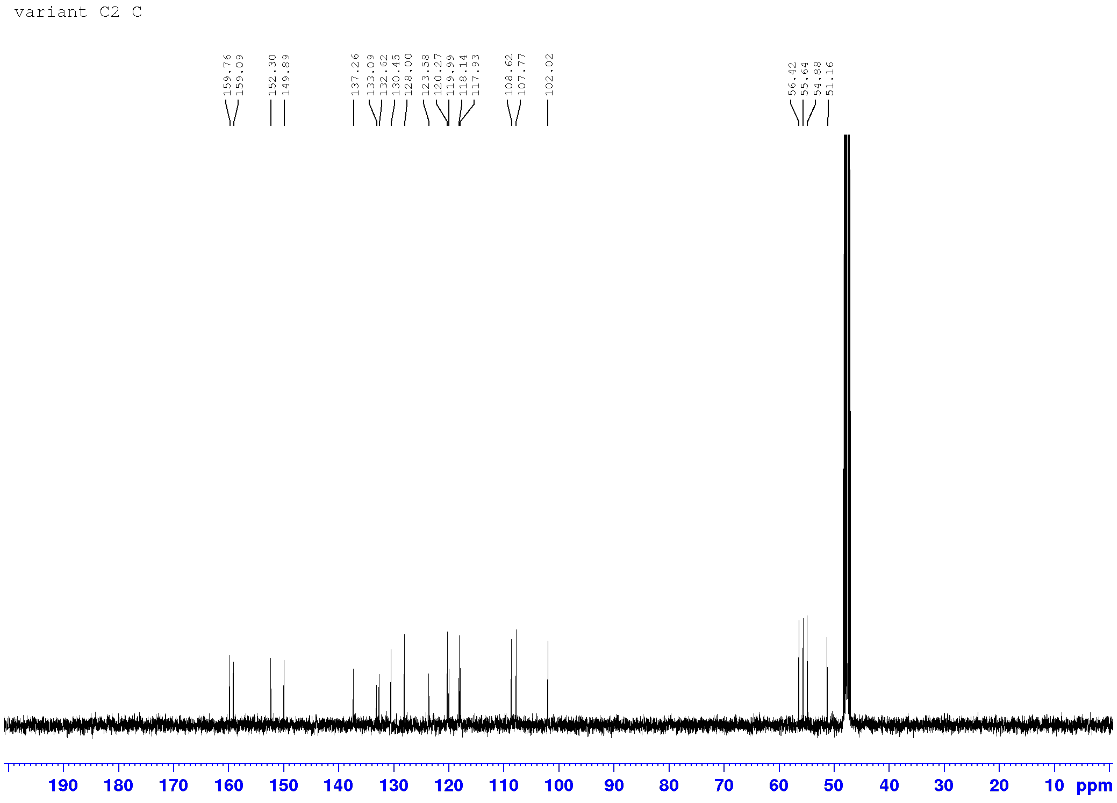
variant C2 C
159.76
159.09
152.30
149.89
137.26
133.09
132.62
130.45
128.00
123.58
120.27
119.99
118.14
117.93
108.62
107.77
102.02
56.42
55.64
54.88
51.16
190 180 170 160 150 140 130 120 110 100 90 80 70 60 50 40 30 20 10 ppm

C3 variant proton

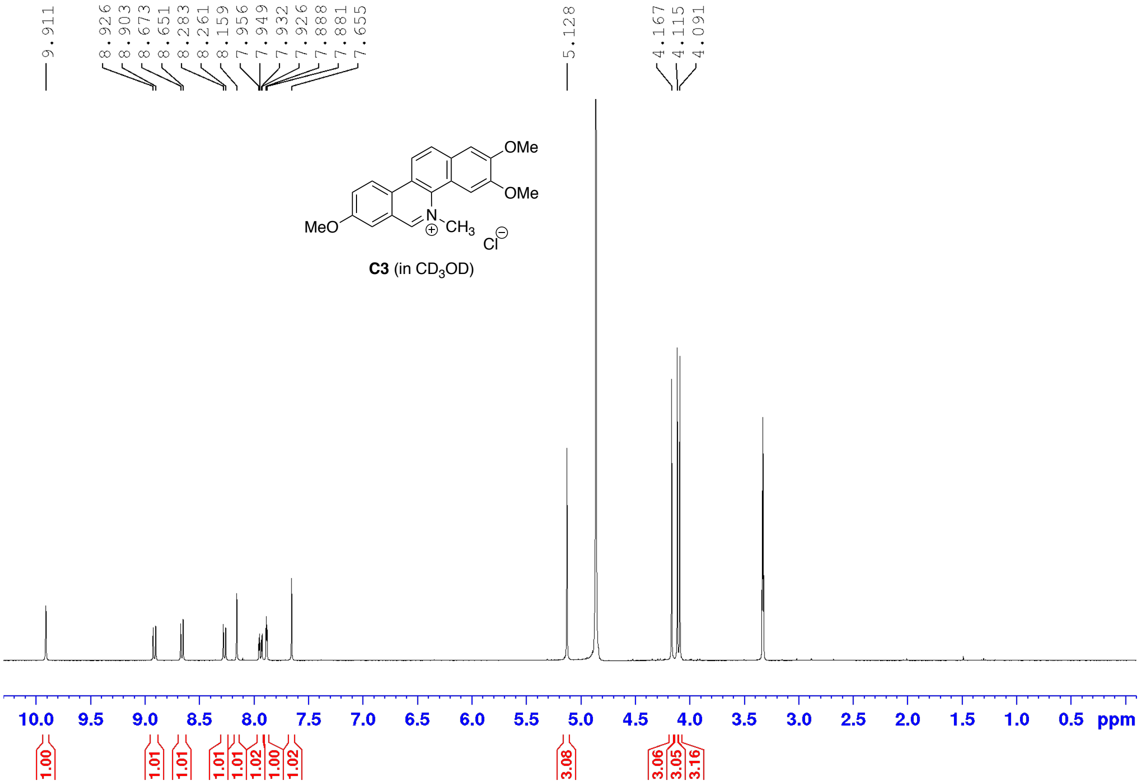

C3 variant carbon

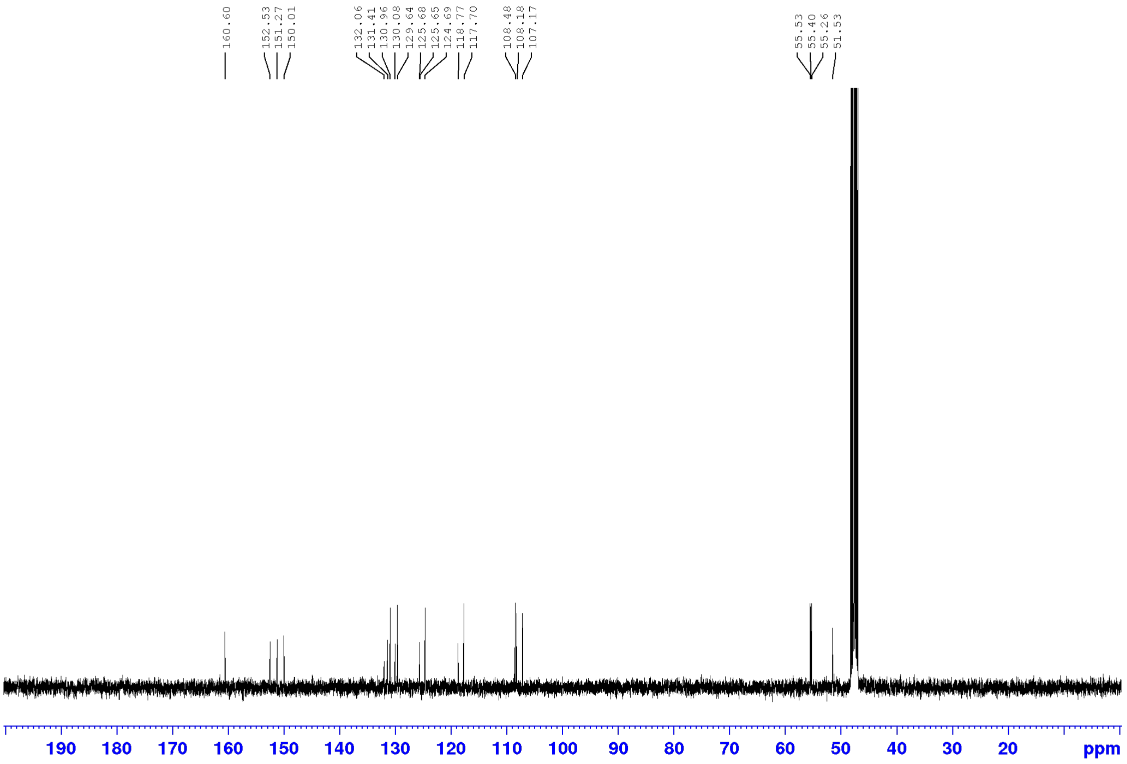

References:

(1) Franceschin, M.; Rossetti, L.; D'Ambrosio, A.; Schirripa, S.; Bianco, A.; Ortaggi, G.; Savino, M.; Schultes, C.; Neidle, S. *Bioorganic & Medicinal Chemistry Letters* **2006**, *16*, 1707–1711. doi:10.1016/j.bmcl.2005.12.001

(2) Liu, H.; Wang, L.; Li, Y.; Liu, J.; An, M.; Zhu, S.; Cao, Y.; Jiang, Z.; Zhao, M.; Cai, Z.; Dai, L.; Ni, T.; Liu, W.; Chen, S.; Wei, C.; Zang, C.; Tian, S.; Yang, J.; Wu, C.; Zhang, D.; Liu, H.; Jiang, Y. *ChemMedChem* **2014**, *9*, 207–216. doi:10.1002/cmdc.201300332

(3) Liao, S.-R.; Zhou, C.-X.; Wu, W.-B.; Ou, T.-M.; Tan, J.-H.; Li, D.; Gu, L.-Q.; Huang, Z.-S. *European Journal of Medicinal Chemistry* **2012**, *53*, 52–63. doi:10.1016/j.ejmech.2012.03.034

(4) Geen, G. R.; Mann, I. S.; Valerie Mullane, M.; McKillop, A. *Tetrahedron* **1998**, *54*, 9875–9894. doi:10.1016/S0040-4020(98)00540-7

(5) Nechepurenko, I. V.; Salakhutdinov, N. F.; Tolstikov, G. A. *Chemistry for Sustainable Development* **2010**, *18*, 1–23

(6) Kadam, S. S.; Maier, L.; Šolomek, T.; Nečas, M.; Šmejkal, K.; Dostál, J.; Sklenář, V.; Marek, R. *J. Phys. Org. Chem.* **2013**, *26*, 814–821. doi:10.1002/poc.3175

(7) Xu, B.; Jiang, X.; Xiong, J.; Lan, J.; Tian, Y.; Zhong, L.; Wang, X.; Xu, N.; Cao, H.; Zhang, W.; Zhang, H.; Hong, X.; Zhan, Y.; Zhang, Y.; Hu, T. *J. Med. Chem.* **2020**, *63*, 5841–5855. doi:10.1021/acs.jmedchem.0c00088

(8) Naruto, S.; Mizuta, H.; Nishimura, H. *Tetrahedron Letters* **1976**, *17*, 1597–1600. doi:10.1016/S0040-4039(01)91626-0

(9) Bekaert, A.; Provot, O.; Rasolojaona, O.; Alami, M.; Brion, J.-D. *Tetrahedron Letters* **2005**, *46*, 4187–4191. doi:10.1016/j.tetlet.2005.04.049

(10) Li, X.; Danishefsky, S. J. *J. Am. Chem. Soc.* **2008**, *130*, 5446–5448. doi:10.1021/ja800612r

(11) Aravindan, N.; Vinayagam, V.; Jeganmohan, M. *Org. Lett.* **2022**, *24*, 5260–5265. doi:10.1021/acs.orglett.2c01734

(12) Bruker AXS. APEX4, 2021

(13) Krause, L.; Herbst-Irmer, R.; Sheldrick, G. M.; Stalke, D. *J Appl Crystallogr* **2015**, *48*, 3–10. doi:10.1107/S1600576714022985

(14) Dolomanov, O. V.; Bourhis, L. J.; Gildea, R. J.; Howard, J. A. K.; Puschmann, H. *J Appl Crystallogr* **2009**, *42*, 339–341. doi:10.1107/S0021889808042726

(15) Sheldrick, G. M. *Acta Crystallogr A Found Adv* **2015**, *71*, 3–8. doi:10.1107/S2053273314026370

(16) Dharmsathaphorn, K.; McRoberts, J. A.; Mandel, K. G.; Tisdale, L. D.; Masui, H. *American Journal of Physiology-Gastrointestinal and Liver Physiology* **1984**, *246*, G204–G208. doi:10.1152/ajpgi.1984.246.2.G204

(17) Bouyer, P. G.; Tang, X.; Weber, C. R.; Shen, L.; Turner, J. R.; Matthews, J. B. *American Journal of Physiology-Gastrointestinal and Liver Physiology* **2013**, *304*, G142–G156. doi:10.1152/ajpgi.00483.2011
